# Fast Synchronization of Ultradian Oscillators Controlled by Delta-Notch Signaling with Cis-Inhibition

**DOI:** 10.1371/journal.pcbi.1003843

**Published:** 2014-10-02

**Authors:** Hendrik B. Tiedemann, Elida Schneltzer, Stefan Zeiser, Wolfgang Wurst, Johannes Beckers, Gerhard K. H. Przemeck, Martin Hrabě de Angelis

**Affiliations:** 1 Institute of Experimental Genetics, Helmholtz Zentrum München - German Research Center for Environmental Health, Neuherberg, Germany; 2 Kinesis Pharma BV, Breda, The Netherlands; 3 Institute of Developmental Genetics, Helmholtz Zentrum München - German Research Center for Environmental Health, Neuherberg, Germany; 4 Technische Universität München, Center of Life and Food Sciences Weihenstephan, Chair of Developmental Genetics, Freising, Germany; 5 Technische Universität München, Center of Life and Food Sciences Weihenstephan, Chair of Experimental Genetics, Freising, Germany; Ecole Normale Supérieure, France

## Abstract

While it is known that a large fraction of vertebrate genes are under the control of a gene regulatory network (GRN) forming a clock with circadian periodicity, shorter period oscillatory genes like the Hairy-enhancer-of split (Hes) genes are discussed mostly in connection with the embryonic process of somitogenesis. They form the core of the somitogenesis-clock, which orchestrates the periodic separation of somites from the presomitic mesoderm (PSM). The formation of sharp boundaries between the blocks of many cells works only when the oscillators in the cells forming the boundary are synchronized. It has been shown experimentally that Delta-Notch (D/N) signaling is responsible for this synchronization. This process has to happen rather fast as a cell experiences at most five oscillations from its ‘birth’ to its incorporation into a somite. Computer simulations describing synchronized oscillators with classical modes of D/N-interaction have difficulties to achieve synchronization in an appropriate time. One approach to solving this problem of modeling fast synchronization in the PSM was the consideration of cell movements. Here we show that fast synchronization of Hes-type oscillators can be achieved without cell movements by including D/N cis-inhibition, wherein the mutual interaction of DELTA and NOTCH in the same cell leads to a titration of ligand against receptor so that only one sort of molecule prevails. Consequently, the symmetry between sender and receiver is partially broken and one cell becomes preferentially sender or receiver at a given moment, which leads to faster entrainment of oscillators. Although not yet confirmed by experiment, the proposed mechanism of enhanced synchronization of mesenchymal cells in the PSM would be a new distinct developmental mechanism employing D/N cis-inhibition. Consequently, the way in which Delta-Notch signaling was modeled so far should be carefully reconsidered.

## Author Summary

During vertebrate embryonic development, the segmented structure of the axial skeleton is laid down by the process of somitogenesis. Periodically, blocks of cells separate at the anterior end of a mesenchymal tissue (PSM) on either side of the neural tube and develop later into spinal vertebrae. Cellular oscillators operating in each cell of the PSM control this process. Their synchronization is essential, and is effected by direct cell-to-cell signaling of the Delta/Notch (D/N) pathway. To better understand the regulation of the genes involved, we employ computer modeling. In this case, the fast synchronization of the oscillators represents a special challenging and worked so far only by the integration of cell movements. Now, we have succeeded in accelerating the synchronization for the first time without cell movements by the interposition of the novel mechanism of intracellular reciprocal inhibition termed D/N *cis*-inhibition into our computer simulations.

## Introduction

Adaption to the day-and-night-cycle on earth provides an evolutionary advantage for organisms that can adjust their gene activity to this 24-hour rhythm. Therefore many metabolic processes show a circadian periodicity because they are all controlled by a GRN forming the so-called circadian clock [1]. Shorter period oscillators are called ultradian [2]. Some play an important role in the embryonic process of somitogenesis, where the vertebrae-precursors, the somites, bud off with a species-specific periodicity at the anterior end from a mesenchymal tissue on both sides of the notochord, the presomitic mesoderm. For mice this period is with two hours much shorter than circadian.

The core of the somitogenesis clock, first simulated in a computer model by Meinhardt [3], is set up in probably all vertebrate species by the Hes/Hairy/her gene families [4], which are negative feedback oscillators. A short decay time for the gene products together with a long enough time delay between gene expression and binding of the protein on its own gene promoter results in oscillatory gene expression. In mice the *Hes1*, *Hes5* and *Hes7* genes (and many others connected to them in an intricate network) were found to oscillate in the PSM [5]. *Hes1*, which also oscillates in neural progenitors [6], could be stimulated to oscillate with a two-hour period *in vitro* in fibroblasts, neuroblasts, myoblasts and other cell types [7]. In the anterior unsegmented PSM of mice, also called wave zone, *Hes7* needs additional activation by D/N signaling to maintain oscillatory gene expression [8]. The D/N pathway works by juxtacrine signaling: Membrane-anchored DELTA or JAGGED ligands of a signal-sending cell bind to NOTCH receptors embedded in the cell membrane of an adjacent cell. This induces a proteolytic cleavage of the NOTCH receptor and releases the intracellular domain of NOTCH (NICD) into the cytoplasm, which then moves into the nucleus where it serves together with various co-factors as transcription regulator and activates, among others, the *Hes1*/7 genes [9]. These events finally lead to a moving wave of NICD from posterior to anterior in the PSM. We proposed in our 2012 model that this wave is generated by the action of the posterior-to-anterior gradients of FGF8 and WNT3a on decay rates of the core oscillator consisting of D/N and Hes7 [10]. When the NICD wave comes to a halt in the anterior PSM, NICD determines together with TBX6 the expression of *Mesp2* that induces the formation of a border between a forming somite and the remaining PSM [11].

Another important function of D/N signaling in somitogenesis is synchronization of the cellular oscillators in the PSM [12], [13], which requires cell-cell contact [14]. Without this synchronization somite formation is severely disturbed [14]. The synchronization of cellular oscillators was also examined theoretically, mostly for the zebrafish *her1*/7 system. Using delay differential equations, D/N signaling was able to synchronize two cells [15] or a row of cells [16]. However, if this system is expanded to 2-dimensional arrays of cells the short-range interaction of D/N causes different domains to be synchronized to different phases and no domain is able to conquer the others [17], [18]. It was shown for zebrafish and chicken that cell movements in the posterior part of the PSM occur depending on the concentration of FGF8 [19], [20]. Uriu et al. included these movements into simulations of the zebrafish PSM and could thereby demonstrate a much better synchronization of the *her* oscillators [18]. Later, this theory was extended to find an optimal rate for cell movements and to describe the effect of gradual recovery of intercellular coupling experienced by a cell after movement [21].

All these models assumed direct interaction between DLL1 and NOTCH1 when they are positioned in membranes of adjacent cells. However, Delta-ligand and Notch-receptor molecules can also interact within the endoplasmatic reticulum (ER) or cell membrane of the same cell [22], [23], which would lead to a fast clearance of the intracellular dimer. This mechanism, where Delta and Notch inhibit each other in the same cell, was therefore termed D/N-cis-inhibition. For example, D/N cis-inhibition is able to generate mutually exclusive signaling states in a mammalian cell-culture system [24]. Applied to computer simulations, D/N cis-inhibition leads to sharper and faster boundary formation during development of the *Drosophila* wing vein system and improves the equidistant distribution of bristle precursor cells by lateral inhibition [25].

Here, we propose another beneficial effect of D/N cis-inhibition: It accelerates in computer simulations the synchronization of D/N coupled ultradian oscillators and extends the parameter range wherein synchronization is possible without taking cell movements into account. Although experimentally not yet confirmed, the proposed mechanism of enhanced synchronization of mesenchymal cells in the PSM would be the third distinct developmental mechanism employing D/N cis-inhibition. Consequently, the way in which Delta-Notch signaling was modeled so far should be carefully reconsidered.

## Results

### The model

**Figure 1:**
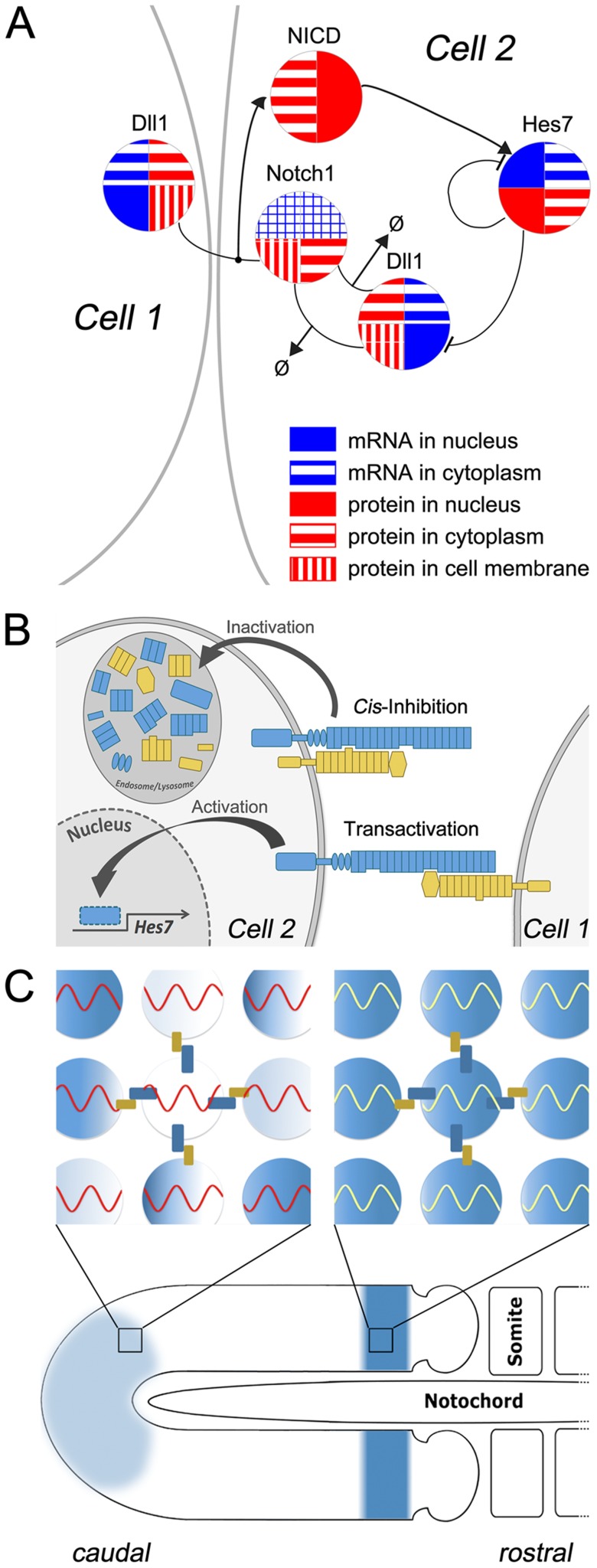
Synchronization of gene expression in somitogenesis by Delta/Notch cis-inhibition. Panel **A** shows our reaction scheme depicting the gene regulatory network. It is sketched for one cell (right) and part of a neighboring cell (left) showing those reactions that involve ligand-receptor interactions in D/N signaling and the *Hes7* oscillator. Gradient forming genes in the PSM like Fgf8, Wnt3a, and Tbx6 are not shown. Each circular area represents one gene, mRNA and protein are color coded blue and red, respectively. For fast changing gene products we simulate the transport between cellular compartments explicitly, which is indicated by subdivided circle half-areas. Activating or repressing arrows represent regulatory interactions. Degradation or decay reactions are symbolized by arrows to the empty-set symbol. For clarity, we omit in the scheme all species decays except for the D/N cis-interaction, which is assumed to lead to a fast decay of the intracellular D/N complex. Panel **B** is a sketch showing D/N interactions. D/N-transactivation (lower part of the panel): DLL1 ligand (yellow) on cell 1 binds to NOTCH1 receptor in the membrane of cell 2, whereupon the NOTCH1 intracellular domain is cleaved off, moves into the cell nucleus and activates the *Hes7* promoter. D/N cis-inhibition (upper part of the panel): We assume an excess of Notch1 in cell 2. DLL1 molecules in the membrane of cell 2 bind NOTCH1 in the same cell and are inactivated after endocytosis to a lysosome. Panel **C** shows a schematic drawing of the growing PSM: in the anterior region of the PSM (right) cells are synchronized as shown in the blow-up of a small rectangle of the PSM of this region, while in the tail bud cells are not synchronized i.e. out of phase as shown in the blow-up of the left small rectangle. Cells in the blow-ups are coupled by D/N signaling (small yellow and blue bars on the surface of the central cell). Connections are shown only for the central cell.

We employ the same cell- and gene-based simulation tool as described in [10]. The GRN incorporated in each virtual cell consisting of Hes7, Delta1, Notch1, is shown in Fig. 1 A and in an advanced version including also Lfng in Fig. S1. Oscillations are generated by a negative feedback of HES7 onto the *Hes7* promoter with delay, which is simulated by the transport of proteins and mRNAs between the nucleus and cytoplasm similar to the transport-model by Uriu et al. [18]. Furthermore, the Hes7 oscillators are coupled by D/N signaling and we assume that HES7 acts on the *Dll1* promoter as it was shown for HES1 [26]. The DLL1 ligand and NOTCH1 receptor are modeled with two compartments for the proteins (cytoplasm and membrane) and for their mRNAs (cytoplasm and nucleus): Since we assume that *Notch1* expression does not oscillate we do not differentiate between nucleus and cytoplasm in this case, because a mathematical description without delay for the mRNA is sufficient. Our model is designed for the simulation of mouse development, therefore the reaction rates are taken from literature or if not available adjusted to reproduce a mouse specific oscillation period of around 2–3 hours. However, our program allows other oscillation periods by simply rescaling all reaction rates in the differential equations – except those in the denominators – via its graphical user interface, which is equivalent to a rescaling of time. In addition to the reaction of DLL1 and NOTCH1 between neighboring cells leading to the release of NICD as transcription co-factor (trans-activation), this work also considers the reaction of NOTCH1 and DLL1 in the membrane and cytoplasm of the same cell (cis-interaction), which leads to their immediate decay – shown graphically in Fig. 1 B. So, the titration of one membrane protein against the other in each cell leads to an excess of either the ligand or the receptor and consequently determines whether the cell acts as a sender or receiver.

### Adding noise to the system

Contrary to our previous work [10], where every cell started with the same initial concentration values and received after mitosis the concentration values of its mother cell at their respective oscillation phases, here, all cells start with random initial values. To avoid that the cells start too far away from their limit cycle we add random values between zero and one multiplied to each of the initial concentration values used in [10] and scaled with a percentage value that gives a simple measure for the initial noise. For instance, 200% noise means: to each concentration its doubled value is added multiplied by a random number taken from the interval between zero and one.

### A simple measure for synchronization

Our program allows for real time observation of the simulation, so synchronization can be easily observed by visual inspection. However, to get a quantitative measure for synchronization we introduced a simple correlation function that falls to zero when perfect synchronization is achieved and shows oscillatory behavior otherwise. In the case of anti-synchronization, the values of the correlation function display negative oscillations.
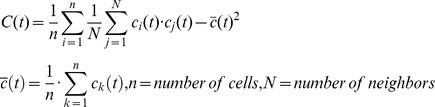
Here 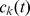 stands for any concentration value of a gene product in cell k (or i or j) at time t and 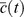 is the average concentration value. For each cell with index i its concentration is multiplied with the average concentration of its neighboring cells with index j, where N is the number of neighboring cells. A rectangular arrangement of cells results in N = 2 for 1 dimension, N = 4 for 2 dimensions, and N = 6 for 3 dimensions. For cells situated on an edge or corner the number of neighbors is reduced, i.e. we use not periodic boundary conditions in our simulations. So N in the formula above depends on cell index i, but we suppress this dependence to simplify the notation. Furthermore, the user can define an extended neighborhood, which means that e.g. in 2 dimensions the diagonal adjacent cells are counted as neighbors. If all cells are synchronized, *c_i_*(*t*) and *c_j_*(*t*) have the same value, which is equal to the average value. So, the difference in the first formula will become zero. For the evaluation of the correlation function we used *Hes7* mRNA concentration in the cytoplasm if not stated otherwise.

**Figure 2:**
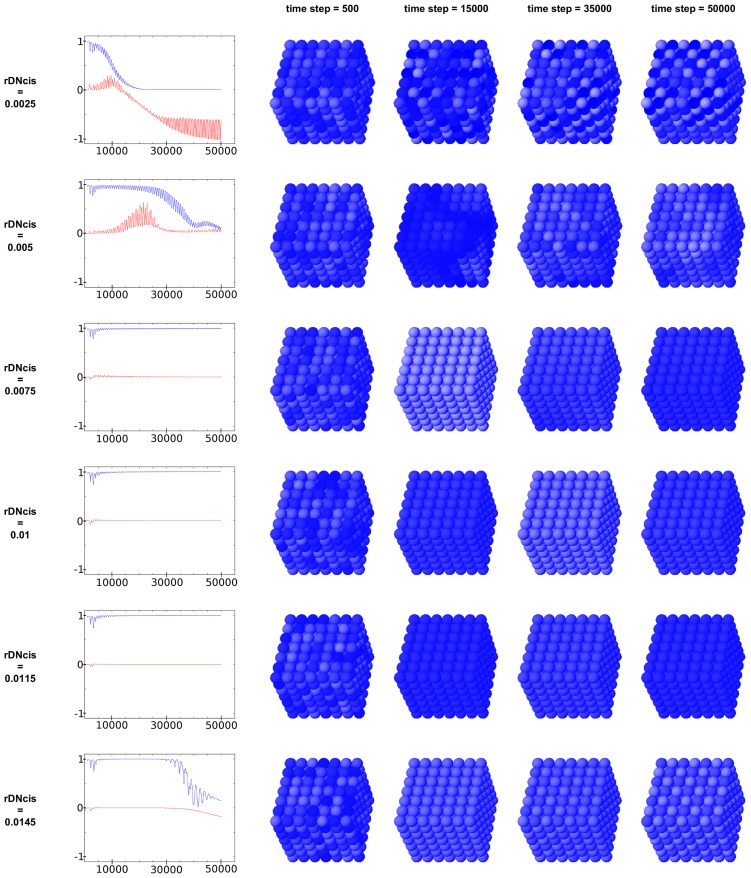
Virtual expression patterns for *Hes7* mRNA in simulation runs with different cis-inhibition values. Snapshots are taken at 500, 15000, 35000, 50000 time steps (1 time step = 0.1 min) after simulation start for a 7×7×7 cell cube for different D/N cis-inhibition strengths. In all cases 100% noise was added at the start of the simulation. On the left side the time course of the correlation function C(t) (red curve) and the synchronization measure R (blue curve) is shown.(Abscissa showing time measured in time steps.)

Although the correlation function uses only information about neighboring cells, it shows us synchronization by dropping to zero, because if each cell is synchronous to its neighbor, all cells are synchronized. Compared to the R-synchronization measure (defined in the supplementary material Text S1), which goes to one for perfect synchronization, the advantage of the correlation function C(t) is the observation that it becomes negative, if the configuration becomes anti-synchronized, i.e. one observes a salt-and-pepper pattern, which can be oscillating or not. See Fig. 2 first and last row for an example for each case.

In our search for parameter values resulting in fast synchronization we observed in the case without D/N cis-inhibition that parameters that allowed for fast synchronization made the system unstable against anti-synchronization. After a period of almost perfect synchronization with C(t) almost exactly zero the system drifts slowly into an oscillating salt-and-pepper pattern with the difference between neighboring cells becoming ever larger. Unfortunately, the faster the synchronization, the shorter the duration of synchronized behavior before reverting into the anti-synchronized state. Because the correlation function allows us to see this behavior before it becomes visible by eye, it is very useful for interactively searching for parameters providing for fast synchronization.

### Effect of cis-inhibition

The effect of D/N cis-inhibition on synchronization of a 7×7×7 cell cube with 100% noise added is shown in Fig. 2, where simulation snapshots are displayed for increasing strengths of D/N cis-inhibition. Clearly, D/N cis-inhibition accelerates synchronization, whereas without (see movie S1) or small cis-inhibition the oscillator-system synchronizes badly and turns after some time into an anti-synchronized state, which was already described for a 2-cell [15] and a 2-dimensional system [17] (see also supplementary movie S2 for the case of rDNcis = 0.01). For intermediate (0.005) values of D/N cis-inhibition one observes incomplete synchronization. Large parts of the cube are synchronous but in different phases to each other so that ‘waves’ of expression moving over the cube volume can be observed. Increasing the D/N cis-inhibition strength leads to complete and ever faster synchronization with the best result achieved for 0.0115. However, increasing D/N cis-inhibition further leads to a progressive damping of the oscillations. This non-oscillating state then turns slowly into a static salt-and-pepper pattern. So in this case we get the classic lateral inhibition case without oscillations.

### Effect of dimensionality and size

**Figure 3:**
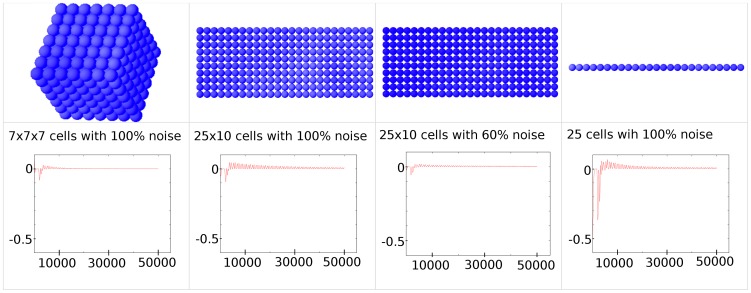
Virtual expression patterns for *Hes7* mRNA for systems of different dimensionality. Snapshots are taken at time point 680 min. At the start of the simulation 100% noise was added. The time course of our correlation function is displayed below ending at 50000 time steps equivalent to 5000 min, which shows how the different systems approach the synchronized state (Correlation function = 0). (Abscissa showing time measured in time steps.)

Simulation snapshots and the time course of our correlation function for systems with different dimensions are shown in Fig. 3. Compared to the 3-dimensional simulation with a 7×7×7 cube of interacting cells, the synchronization of a 2-dimensional array of cells is slower and deviations from perfect synchronization are larger. Only if one reduces the noise amplitude to 60%, the initial deviations in the correlation function are comparable, but synchronization is still slower. A similar effect is observed for a 1-dimensional chain of cells. This can be explained by the nature of our model, where the effect of D/N signaling in the receiving cell is averaged over the number of its neighbors due to practical reasons. This has the advantage that one does not have to change all parameters in the network when dimensionality of the system is changed. Consequently, the noise one cell receives in D/N signaling reduces with the number of its neighbors because fluctuations are cancelled out better in summation with more neighboring cells sending noisy signals. This effect is also demonstrated in Fig. S2, where a 3-dimensional array with 6 neighbors per cell gives comparable results to a 2 dimensional array with 8 neighbors per cell. Likewise, we analyzed the influence of cell number, i.e. the volume of a cell array, on synchronization and compared cubes with a length of 5, 7, 9, 11, and 14 cells (Fig. S3). While at the beginning the correlation functions vary due to the randomly chosen initial values, they decay in the further course of the simulation to very small values with a similar behavior. The same behavior can be observed also for the R-synchronization-measure, which quickly reaches values very near 1, indicating very good synchronization, independently of the size of the cell cube.

### Effect of parameter variation

To explore the robustness of the system and the speed of D/N-mediated synchronization, with and without cis-inhibition, we performed an extensive scan over all parameters in a simple two-cell system. As expected, D/N cis-inhibition provides for faster synchronization of cells over a wide parameter range, independent from the chosen initial concentration values (for details see supplemental text S1). There are also parameter ranges where synchronization is not achieved with D/N cis-inhibition, if one looks at the R-synchronization measure. However, if one looks at the concentration time course behavior one sees that this downward trend of the R-function results from a progressive damping of the oscillations if one increases the Hes7 mRNA or protein decay rates more than ten percent, for instance.

The influence of the system parameters on the amplitude (minimal and maximal cytoplasmic HES7 expression) of the cellular oscillator is shown in Fig. 3 of the supplemental text S1. One can observe the strong dependence of the oscillator amplitude on *Hes7* mRNA and protein decay rate, for instance, and that the cis-inhibition strength rDNcis abolishes the oscillation if it increases beyond 0.014, as already seen in Fig. 2.

To examine the robustness of the system further we generated 40 parameter sets by randomly varying all production, transport, and decay rates within a range of plus-minus ten percent around our standard parameters and tested these parameters sets in a cube with an edge length of 7 cells with 100% initial noise added. 16 of the random parameter sets resulted in damped oscillation and of the 24 undamped oscillating systems 21 showed complete synchronization. Only for three parameter sets synchronization was not complete. Instead, expression waves were generated. Results for all oscillating parameter sets are shown Fig. S4. The input files for running simulations with the different parameter sets are supplied in the supplemental material as file S1 (Config-files.tar.gz).

### Results for an extended GRN including Lfng

In our previous work on boundary formation in the PSM of mouse [10] we postulated a positive action of LFNG on D/N signaling. Likewise, we have extended our minimal model by *Lfng*, which is controlled by HES7 (Fig. S1). Here, the parameters chosen for the relative contributions of unaided D/N signaling and D/N interaction with LFNG-action have to meet two demands: (i) they should allow fast synchronization with D/N cis-inhibition, and (ii) they should reproduce the diminished oscillation amplitude observed experimentally in the mouse PSM when *Lfng* is non-functional [27]. These demands are fulfilled when we set the ratio of unaided to LFNG-promoted D/N reaction to about 1∶4 (Fig. S5).

### D/N cis-inhibition in a simple model of somitogenesis

**Figure 4:**
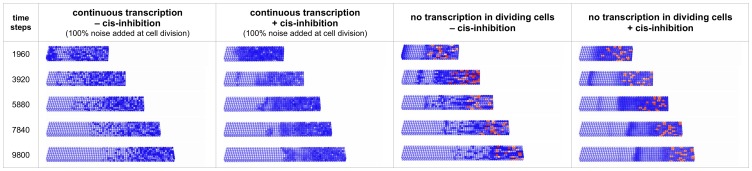
Snapshots of virtual expression patterns for *Hes7* mRNA in simulations of the growing PSM. The posterior-to-anterior FGF8 gradient is coupled to the HES7 decay. One time step equals 0.1 minute. From left to right shown are the cases of 100% noise added during division of cells in the growth zone of the PSM without (shown also in movie S3) and with (shown also in movie S4) D/N cis-inhibition. Snapshots are also displayed for simulation runs wherein the disturbance of oscillator consonance is caused by shutting down the transcription of the core oscillator genes during mitosis. Shown are the cases of 20 min shutdown of the transcription during cell division in the growth zone of the PSM without (shown also in movie S6) and with (shown also in movie S5) D/N cis-inhibition. Cells are colored orange in the simulations as long as transcription of their genes is shut down.

So far, all discussions on synchronization of ultradian oscillators by D/N signaling examined the static case, i.e. a non-growing tissue. However, a real test for synchronization would be a growing tissue, for example, the tail bud during somitogenesis (Fig. 1 C). Therefore, we implemented D/N cis-inhibition in one of our models of somitogenesis, which is characterized by a growing tissue and a posterior-to-anterior FGF8 gradient controlling HES7 degradation [10]. When daughter cells inherit the concentration values of their mother cells and a 100 percent noise is added, we observed a clear difference between simulations without (movie S3) and with (movie S4) D/N cis-inhibition (Fig. 4). However, even with cis-inhibition instabilities have arisen after the fourth oscillation. To allow for more realistic noise-affected gene expression, we simulated mitosis by developing a model in which the dividing cells in the growth zone of the PSM shut off transcription, which consequently disturbs *Hes7* expression waves after two oscillations even when the cells started synchronized at the beginning of the simulation. Furthermore, we allowed diagonal neighbors to signal via D/N. For a mitosis phase of 20 min, D/N cis-inhibition was able to maintain phase coherence reasonably well (movie S5), whereas without D/N cis-inhibition (movie S6) the initial order was lost after two oscillation periods (Fig. 4).

In summary, our results demonstrate that the inclusion of D/N cis-inhibition in the formulation of the model brings about a decisive improvement in the ability of D/N signaling to synchronize cellular oscillators. This is achieved not only for a specially chosen set of parameters, but a wide range of model parameters.

## Discussion

### General remarks

The aim of our modeling work in somitogenesis is to explain how the various expression waves in the mouse PSM are generated, why they slow down when they are nearing the anterior end of the unsegmented PSM, and how the boundary between the PSM and the next forming somite is formed. In our previous paper [10] we were concerned with the generation of the NICD wave and why it stops, because together with the TBX6 and FGF8 gradients NICD induces *Mesp2*, which is critically important for boundary formation. Our hypothesis for the generation of the NICD wave was that the WNT3A and/or FGF8 gradients in the PSM influence an intracellular process of the core oscillator consisting of Hes7 and D/N thereby slowing the oscillator down when it gets out of the range of the gradients. Therefore, we modeled the core oscillator as a transport model with the most important cellular compartments (nucleus, cytoplasm, and membrane) and processes like transcription, translation and transport and allowed a possible coupling of each gradient to each cellular process. Furthermore, we included as many measurable parameters and especially promoter information as we could find in the literature (which is unfortunately rather sparse). However, with plausible assumption one can generate at least the qualitative behavior with its characteristic expression pattern rather well. The drawback of our method is that one cannot sample the multidimensional parameter space. However, if new information becomes available, one can feed it directly into our model.

In our 2012 paper [10] we had excluded the synchronization problem. Cells started synchronized and stayed so, because during proliferation daughter cells inherited the oscillatory phase of their mother cells. However, as NICD and D/N-signaling are widely held to be responsible for the maintenance of oscillations and synchronization of wave formation and in creating boundaries in space as the waves come to rest, one should work towards a comprehensive model including synchronization.

### The role of D/N in synchronization

In somitogenesis the formation of sharp boundaries between the block of cells forming a new pair of somites and the remaining PSM works only when gene expression in the cells forming the boundary is synchronized. It has been shown experimentally that D/N signaling is responsible for this synchronization. The species-specific periodicity of somitogenesis is controlled by cellular oscillators, in mouse most probably by the negative feedback oscillator *Hes7*. The synchronization has to happen rather fast as a cell experiences about five oscillations from its birth to its incorporation into a somite [28]. Computer simulations describing oscillators coupled by classical modes of D/N-interaction failed so far to achieve synchronization in an appropriate time approach except by introducing cell movements in simulations. Here we show that fast synchronization of Hes-type oscillators can be achieved without cell movements by including the process of D/N cis-inhibition.

While in conventional models of D/N synchronized oscillations each cell is sender as well as receiver of D/N-signaling because DELTA ligands as well as NOTCH receptors are active in the membrane of the cell, in a system with perfect cis-inhibition i.e. perfect titration of DELTA against NOTCH or vice versa, a cell is either sender or receiver. That means that a cell with DELTA excess – an information sender - can enforce a change in NICD controlled gene expression in a neighboring receiver cell, i.e. with NOTCH excess, as fast as intrinsic NICD processes allow in the receiver cell. If Delta expression is oscillatory – as in our model - the sender cell could go into receiver mode if Delta expression is low. So other cells could influence/synchronize this cell. In this manner, fast synchronization could be achieved despite the fact that the cell-interaction is still local (even if one considers communication by cytonemes as observed in zebrafish [29]). This does not exclude the possibility that for very large volumes the locality of cell-cell-communication leads to domains synchronized to different phases, but for realistic numbers of cells the above acceleration of synchronization could be sufficient i.e. fast enough.

However, for D/N synchronization of *Hes7*-oscillators the considerations shown above are too simplified, as a cell cannot be only sender, i.e. have any active NOTCH in its membrane. This is so because *Hes7* activation relies on NICD and in our model of the core oscillator HES7 suppresses *Dll1* expression leading to the oscillatory DELTA expression mentioned above. Consequently, a sender-only cell would have no interesting message to send. So a perfect titration of NOTCH against DELTA is not desirable. There has to be an optimum value of cis-inhibition. If this value is surpassed oscillations are damped and die out. This was shown in Fig. 2.

### Assumptions of the model

At least for mouse, there is strong evidence that the *Hes7* gene oscillates by negative feedback of its protein on its own promoter, thereby serving as the core oscillator of the somitogenesis clock [30]–[32]. Furthermore, promoter analysis revealed that *Hes7* is induced by D/N signaling [33]. The NOTCH modifying gene *Lfng* is also induced by D/N-signaling and oscillates in the PSM because its expression is inhibited by HES7 [33].

The fact that D/N-signaling is required for the synchronization of ultradian oscillators in the PSM was shown for zebrafish [16], [34] in experiments with single cell resolution. Because it is not easy to separate the induction of oscillation and synchronization in mouse on the cellular level, Okubo et al. used chimeric embryos composed of wild-type and *Dll1*-null cells to demonstrate that D/N-signaling is responsible for the synchronization of oscillations in the PSM also in mouse [13]. To clarify the role of *Lfng* in the somitogenesis clock, Okubo et al. also analyzed *Lfng* chimeric embryos and used Notch signal reporter assays in a co-culture system [13]. As interpretation of the results they proposed a novel, in this form not yet described action of LFNG on DLL1. The knockout of *Lfng* resulted in an enhanced activity of NICD in the PSM, which indicates that LFNG might affect NOTCH1 and DLL1 negatively. Okubo et al. also demonstrated that the synchronization of cellular oscillators was proportional to the number of *Dll1* expressing (wild-type) cells in chimeric embryos, which confirmed that D/N synchronizes *Hes7* oscillations in the PSM. Similarly, using *Lfng* chimeric embryos, they showed that LFNG seems to be required for this synchronization. Interestingly, computer simulations that integrated the proposed effect of LFNG on NOTCH1 and DLL1 showed fast oscillator synchronization and were able to reproduce their experimental findings [13]. In their model the *Hes7* oscillator in every cell is coupled to neighboring cells via LFNG, which is itself driven by HES7 oscillations and regulates the intracellular coupling by inhibition of both NOTCH1 and DLL1 activity in the same cell. Thus, LFNG not only represses D/N signaling inside the LFNG expressing cell by modifying NOTCH1 cell-autonomously, but also represses D/N signaling between neighboring cells by also modifying the DLL1 ligand. In short, in their model the output of the *Hes7* oscillator is coupled to D/N signaling exclusively by the way of LFNG action.

In contrast, in our model we assume that HES7 inhibits *Dll1* expression like Her1/7 inhibits *deltaC* in zebrafish. We will not repeat the extensive discussion provided in our previous publication [10], but strengthen the main arguments, which are that expression of *Dll1* is dynamic in the PSM [35] and that only the orthologs of *Hes7* and *Dll1* are dynamic in the PSM of all vertebrate systems examined so far [36]. For example, *Lfng* expression is constant in zebrafish as well as in medaka [36]. Therefore, we argue for an evolutionary mechanism with a zebrafish-like core oscillator in which LFNG acts only in a modulatory role. Consistent with this notion, NICD expression is still dynamic in *Lfng* deficient mice [37] and *Lfng* is not required for somite formation in the tail bud phase [38]. In this work, we therefore examined the effect of D/N cis-inhibition primarily in a model without modulation of D/N signaling by LFNG.

Quantitative data regarding cell cycle parameters in mouse embryogenesis are sparse. Power and Tam give a value of ca. 30 min for 7.0-day embryos [39].

### Other mechanisms not included in the simulation

When judging about the success or failure of our model with respect to the real facts one should not forget that there may be biological mechanism that are not covered by the model, but could be crucial for the functioning of the synchronization. For example, it was found that *Dll1* mRNA is stabilized during mitosis, by *Elavl1/HuR* in neuroepithelial cells [40]. If similar mechanisms are operative in the growth zone of the PSM, our assumption that mRNA decay rates are constant in time could be too pessimistic. A smaller decay rate during mitosis would very probably diminish the perturbation to oscillations and thereby improve synchronization. Interestingly, a study observing oscillatory expression of a Her1-Venus reporter at single cell resolution in the zebrafish PSM found that *her1* oscillations are linked to mitosis [34]. Therefore, it is possible that cell divisions introduce less noise than our model assumes.

### Comparison to circadian systems

In the hypothalamus of the mammalian brain, 20000 nerve cells function as circadian oscillators and have to be synchronized to function as the master circadian clock of the body [41]. Like ultradian oscillators, these circadian oscillators function by a negative transcription-translation feedback loop and are often also modeled by Goodwin-models (see for example [42], [43] and references therein), but also by delay differential equations or very simple toy models [44]. However, compared to the somitogenesis clock, in the circadian clock there are more interlocking feedback loops [41] and the communication between cells works either by secretion of neuropeptides and/or by direct innervation. So, coupling in the circadian clock is not mediated by communication between directly adjacent cells but by non-local interactions, which probably favors tissue-wide synchronization and prevents the phenomenon of cell territories synchronized to different phases ‘fighting’ for dominance. Furthermore, in circadian clock models the synchronization signal acts positively on the transcription of the clock genes. This is also the case in our model of the ultradian oscillator, where NICD acts as an activator on *Hes7* transcription. However, HES7 represses *Dll1* in the same cell and therefore NICD generation in the adjacent cell. This is the reason why lateral inhibition occurs in the static case or leads to anti-synchrony in the dynamic setting. Another difference concerns the coupling of the synchronization signal to the promoter of the clock feedback loop. In circadian models, this is mostly assumed to be additive, whereas we do not assume an additive but a multiplicative coupling of D/N signaling to the *Hes7* promoter because it was shown that in most of the PSM *Hes7* ceases to oscillate without D/N input. We disregard in our model the fact, that Hes7 is induced by FGF8 in the tailbud [8], which would be an additive coupling to FGF8. It was found in circadian oscillator models that weak oscillators, which are damped without a synchronization signal, synchronize faster [42], [43], [45]. As our *Hes7* oscillator is coupled in ‘AND’ modus to the synchronization signal (NICD), this could possibly be seen as an example of this principle. (It was also found for the circadian clock that the oscillator's radial relaxation time scale and the ratio of synchronization signal to the oscillator amplitude are important for synchronization and oscillator entrainment [44].)

### Conclusion

Contrary to Wang et al. [46] who simulate neural fate decisions in the developing nervous system and proposed that D/N cis-inhibition causes asynchrony between adjacent cells, adding D/N cis-inhibition terms to our model of ultradian oscillators of the Hes/Hairy/her type clearly leads to a faster synchronization. Furthermore, the phenomenon of different regions that are synchronized to different oscillation-phase values, and that one region cannot overwhelm another, can be overcome without cell movements, at least for the non-growing case, by introducing D/N cis-inhibition. Since cis-inhibition allows faster reaction of cells on changes in their neighborhood, cell movement may not be required for all situations in which synchronization is mediated by D/N signaling. We also show that D/N cis-inhibition does not interfere with a proposed mechanism for wave generation in the PSM, in which the control of HES7 degradation by the posterior-to-anterior FGF8 gradient slows down the oscillators as they get out of the range of the gradients by the continuous growth of the PSM. That D/N cis-inhibition does not lead to complete synchronization in the whole PSM, which would resist slowing down, is probably caused by the fact that the slowing down gets appreciable only in the last oscillation a cellular oscillator experiences before being incorporated into a somite [10]. However, ultimately, only experiments can clarify whether D/N cis-inhibition [22], [23] is functional also during somitogenesis.

## Materials and Methods

Download information including a mini manual of the program is provided in supplemental Text S2.

We also supply SBML files describing the system for 2 cells without growth (Text S3 (SBML_DeltaNotchModel_2cells_cis.xml) for the model described in Fig. 1 and Text S4 (SBML_DeltaNotchModel_2cells_cis_lfng.xml) for the model described in Fig. S1).

### General features of the model

To model gene expressions we use essentially the same methodology as described in [17], i.e. a gene- and cell-based simulation program that numerically solves differential equations describing a gene regulatory network and displays the concentration of a selected gene product by color intensity (virtual *in situ* staining) in each cell. For showing the consequences of the gene regulatory network (Fig. 1) we use the same cell- and gene based simulation program as in [10] except that cis-inhibitory interaction-terms in the membrane and cytoplasmic compartment were added. Specifically, we use the same formulas and rate constants as in our previous publication, except the addition of the D/N cis-inhibition terms, different values for Hill coefficient and Hill threshold describing the action of NICD at the *Hes7* promoter, and the LFNG coupling. Furthermore, it is now possible to enlarge the neighborhood of a cell so that also diagonally adjacent cells are treated as interacting neighboring cells. In addition, we take into account that the Hill-coefficient for the action of the NICD complex on the *Hes7* promoter could be higher than 2 because of cooperative effects between the dimer formed of a NICD-Maml1-Rbpj-kappa complex and additional chromatin modifying co-factors. As discussed in [10], we introduce distinct variables for cytoplasmic and nuclear concentrations of proteins and the respective mRNAs. This distinction is made for the oscillatory factors HES1/7, NICD and LFNG, but not for the slow-changing concentrations of protein and mRNA of Notch1. The DLL1 ligand and the NOTCH receptor are modeled with independent variables in the cytoplasm and membrane compartments.

In the somitogenesis model we included only the genes from our previous model [10] that are needed to generate the ‘wave’-pattern i.e. *Dll1, Notch1, Hes7*, *Fgf8, Wnt3a, and Tbx6*, because the downstream genes like *Mesp2, Ripply2* and *Epha4* would function similar as in our 2012 publication [10] except for possible Hill-threshold adjustments.

### The *Hes7* oscillator and D/N signaling

A schematic view of the GRN used in our simulations is depicted in Fig. 1. Its central element is the negative feedback oscillator *Hes7*. By binding to the promoter it inhibits its own production. The *Hes7* promoter also receives input from D/N signaling while we disregard here the contribution of Fgf signaling in the tailbud [8]. In an extended model, HES7 inhibits *Lfng*, which is induced by NICD, and in turn modulates D/N interaction. NICD acts as an activator of *Hes7*. Here, we assume that HES7 inhibits *Dll1* expression. For the mathematical description of the model we use ordinary differential equations. To describe negative feedback oscillators one has to introduce a function describing the repressive action of the gene product on the promoter of its gene. We use Hill functions of the form 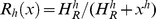 to describe this negative feedback, wherein the Hill-coefficient *h* is a measure for the cooperativity of the repressor binding to the promoter and *H_R_* as well as *H_A_* are the thresholds determining half-inhibition or activation, respectively (see below). For transcription factors binding as homo-dimers we set the Hill coefficient to the value of 2. To describe activating gene action we use analogously Hill functions of the form 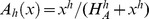


Oscillations start only when there is a delay between gene expression and negative feedback. This is often modeled with direct introduction of delayed arguments into the differential equations specifying the time used for transcribing a gene into mRNA and translating a mRNA into protein, resulting in a so-called delay differential equation system (for an example see [15], [47]). In the following, we specify the differential equations of our gene regulatory network. In all cases the gene indices on the variables written on the right side of the equations are not shown except when the variables refer to other genes. Decay rates are always given in *min^−1^* and concentration values are given in arbitrary units.

### Hes7

The equations below describe the negative feedback oscillator at the core of our GRN:
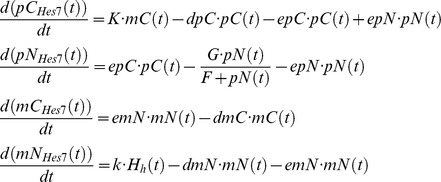
Here *pC*(*t*), *pN*(*t*), *mC*(*t*), and *mN*(*t*) designate concentrations of cytoplasmic protein, nuclear protein, cytoplasmic mRNA, and nuclear mRNA, respectively. The export rates of the protein from cytoplasm to nucleus, from nucleus to cytoplasm, and for the transport of mRNA from nucleus to cytoplasm are chosen as: *epC* = 0.007, *epN* = 0.001, and *emN* = 0.038. Furthermore, *dmC* = 0.067, *dmN* = 0.001, and *dpC* = 0.031 describe the degradation rates for cytoplasmic and nuclear mRNA, and cytoplasmic protein, respectively. Based on experimental evidence, we assume a rather low rate of mRNA degradation in the nucleus for all genes [48]. We suppose saturated protein decay in the nucleus characterized by threshold value *F* = 0.2 and maximum rate *G* = 0.96. The translation rate and the maximal transcription rate are given by *_K_*_ = 1.5_ and *k* = 0.5, respectively. The Hill function 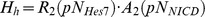 with *H_R_* = 1.0 and *H_A_* = 4.5 describes the negative feedback of HES7 on its own promoter and the control of *Hes7* transcription by the Notch intracellular domain (NICD). The bHLH-transcription factor HES7 binds as dimer to its own promoters thereby inhibiting transcription. The *Hes7* gene contains only one N-box in its promoter [49]. If HES7 would bind also to the so-called E-boxes in the *Hes7* promoter the Hill-coefficient could also be higher [50]. However, Chen et al. have shown that HES7 only binds to the N-box [33], so only one HES7 dimer binds. Therefore we chose a Hill-coefficient of 2. Furthermore, we subsume all interactions with co-factors of HES7 like *Groucho/Tle1* in the basal transcription rate. HES7 is a target of D/N signaling. This entails NICD acting as transcriptional co-factor on the *Hes7* promoter. As it was shown that two complexes comprising NICD, MAML1 and CSL bind as a dimer to the *Hes1* promoter [51] and we assume a similar *Hes7* promoter structure regarding activation by NICD, we also use a Hill-coefficient of 2 or higher for the Hill-function describing the effect of NICD in our simulations.

### Notch intracellular domain (NICD)

NICD is a fragment of the Notch receptor, which is generated after binding of the DLL1 ligand to the NOTCH1 receptor. Ligand binding enables access of proteases to cleavage sites in the intracellular part of NOTCH1 and subsequent transport of NICD from the cytoplasm to the nucleus [52].
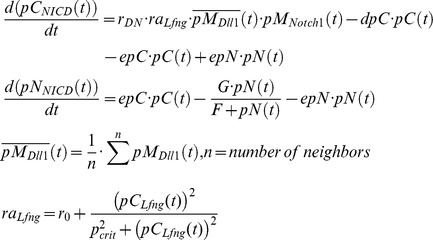
Here, *r_DN_* = 0.05 is the reaction rate between NOTCH1 receptors and the DLL1 ligands on the *n* neighboring cells, while *ra_Lfng_* describes the activation of D/N signaling by LFNG, and *r*_0_ is the reaction rate of DLL1 and NOTCH1 without LFNG action. For the simulations shown here the default value is 0.256. *_pMNotch1_* designates NOTCH membrane protein concentration, *pM_Dll1_* DLL1 protein concentration in the membrane. *epC* = 0.12 and *epN* = 0.6 are the export rates for NICD from the cytoplasm to the nucleus and vice versa, and *dpC* = 0.2 is the NICD decay rate in the cytoplasm. As NICD acts as a co-transcription factor in the nucleus its import rate to the nucleus is chosen larger as the export rate. In the simulations without Lfng in the GRN *ra_Lfng_* is set to 1.

### *Dll1*

At least in the presomitic mesoderm it was demonstrated that *Dll1* expression is dynamic [35]. So the mathematics of negative feedback systems necessitates the use of a transport equation system with at least three equations for oscillatory behavior to be possible [53]. We use two equations for Dll1 mRNA and protein, each in nucleus and cytoplasm, making four differential equations:
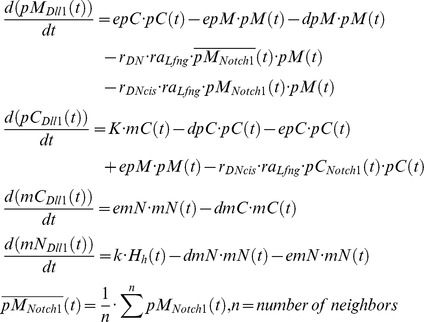
In the PSM *Dll1* is activated directly and indirectly via TBX6 by Wnt signaling [54]. Based on experimental evidence, we assume an additional control by HES7 (see [10] for an extensive discussion). In the spatially constant model system we disregard the control by TBX6 and WNT3A. Therefore, we chose a Hill function of the form 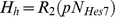, with *H_R_* = 1.0. We chose the rate constants as in [10]: *K* = 1.5, *dpC* = 0.09, *epC* = 0.1, *epM* = 0.1, *dpM* = 0, *dmC* = 0.12, *emN* = 0.09, *dmN* = 0.001 and *k* = 1.25. The rate constant *r_DNcis_* = 0.01 describing D/N cis-inhibition results in fast synchronization.

After binding of one DLL1 molecule in the membrane of one cell to a NOTCH1 receptor in the membrane of a neighboring cell, the intracellular part of NOTCH1 is cleaved off to release NICD. This results in the destruction of the NOTCH1 molecule in this reaction. Therefore, the reaction term is subtracted in the equation describing NOTCH1 in the membrane, while it is added to the NICD equation. Because the DLL1 ligand bound to the extracellular domain of NOTCH1 is endocytosed and probably degraded [55], the same reaction term is subtracted in the equation describing DLL1 in the membrane. We assume that the same applies to the intracellular complex formed by a DELTA and NOTCH molecule.

### *Notch1*

Since we assume *Notch1* expression to be static it suffices to describe its mRNA concentration by one simple equation with a production and decay term i.e. without differentiating between nucleus and cytoplasm.
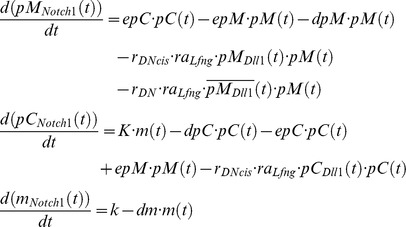
We chose *K* = 1.5, *dpC* = 0.2, *epC* = 0.1, *epM* = 0.0, *dpM* = 0.1, *dm* = 0.02, and *k* = 0.5 for the rate constants.

### *Lfng*

The differential equation system for Lfng has essentially the same structure as the one for *Hes7*, except that HES7 exerts a repressive influence on the *Lfng* promoter while NICD activates it. This is described by the Hill function 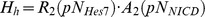 with *H_R_* = 1.0 and *H_A_* = 4.5.
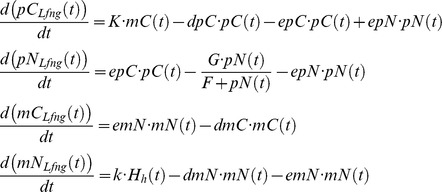
Here *pC*(*t*), *pN*(*t*), *mC*(*t*), and *mN*(*t*) designate concentrations of cytoplasmic protein, nuclear protein, cytoplasmic mRNA, and nuclear mRNA, respectively. The export rates of the protein from cytoplasm to nucleus, from nucleus to cytoplasm, and for the transport of mRNA from nucleus to cytoplasm are chosen as: *epC* = 0.007, *epN* = 0.001, and *emN* = 0.038. Furthermore, *dmC* = 0.067, *dmN* = 0.001, and *dpC* = 0.031 describe the degradation rates for cytoplasmic and nuclear mRNA, and cytoplasmic protein, respectively. Again we suppose saturated protein decay in the nucleus characterized by threshold value *F* = 0.2 and maximum rate *G* = 0.96. The translation rate and the maximal transcription rate are given by *K* = 1.5 and *k* = 0.5, respectively.

### D/N cis-inhibition in a simulation of somitogenesis

For the modeling of growth and geometry in the growing PSM we refer to [10]. We also use the same parameters and equations for the *Wnt3a, Tbx6, and Fgf8* genes described therein. *Dll1* and *Notch1* induction by WNT3A and TBX6, i.e., their corresponding Hill functions, are also chosen as in [10].

Noise is introduced by shutting off transcription only for Hes7, Dll1, Notch1, and Lfng i.e., not for the gradient generating genes, because in our model there is no way this noise could be corrected by D/N signaling. To include this, one would have to simulate a full model of the Wnt3a and Fgf8 pathway with genes like *Nkd1* or *Dusp4* and others, which exert a negative feedback on their respective pathways and are known to be controlled by D/N signaling [56].

#### Technical remark

To run the program in somitogenesis plus mitosis mode one has set the noise level to zero, chose the mitosis time (in time steps) during which transcription is shut off and for which genes. If the simulation runs to slow one should reduce the number of cells in a row.

## Supporting Information

Figure S1 **The gene regulatory network with the gene** ***Lfng*** **included.** Color codes like in Fig. 1.
(TIF)

Figure S2 **Virtual expression patterns for** ***Hes7*** **mRNA for systems of different dimensionality.** Virtual expression patterns for *Hes7* mRNA (top row) at time point 680 min for systems of different dimensionality. At the bottom the time course of our correlation function is displayed, which shows how the different systems approach the synchronized state (Correlation function = 0). At the left side a 3-dimensional system with 6 neighboring cells, at the right a 2-dimensional system with 8 neighboring cells (light blue) signaling to the red cell. (100% ‘noise’ added).
(TIF)

Figure S3 **Time course of synchronization for different volumes.** In the bottom panel time courses of correlation functions C(t) (red curve) and synchronization measures R (blue curve) for systems of different volumes (with 100% noise added) are shown. The top panel shows *Hes7* mRNA expression in the cubes with edge lengths of 5, 7, 9, 11, and 14 cells, respectively, at the end of each simulation run.
(TIF)

Figure S4 **Virtual expression patterns for** ***Hes7*** **mRNA resulting from different parameter sets.** Snapshots of *Hes7* mRNA at 500, 5000, 15000, 35000, 50000 time steps (1 time step = 0.1 min) after simulation start for a 7×7×7 cell cube for different parameter sets generate by randomly changing all transport-, production-, and decay-rates within boundaries of plus-minus ten percent of our default values. In all cases 100% noise was added at the start of the simulation. On the left side the time course of the correlation function C(t) (red curve) is shown. Parameter sets #10, #20 and #24 s result in expression patterns which don't synchronize, but show wave like behavior.
(TIF)

Figure S5 **Simulation with the GRN shown in Figure S2 in each cell.** Virtual expression patterns for *Hes7* mRNA at 39, 220, 440, 520, 720 min after simulation start for a 7×7×7 cell cube. Top row with D/N cis-inhibition, bottom row without D/N cis-inhibition. Both cases with 150% noise added.
(TIF)

File S1 **Configuation files.** The zipped archive file includes the configuration files for running the model with the parameter sets resulting in the model behavior shown in Fig. S4.
(GZ) Click here for additional data file.

Movie S1 **A 7×7×7 cell cube started with 150% noise added, without active Delta-Notch cis-inhibition.** Shown is the cytoplasmic *Hes7* mRNA concentration.
(MOV)

Movie S2 **A 7×7×7 cell cube started with 150% noise added, with active Delta-Notch cis-inhibition.** Shown is the cytoplasmic *Hes7* mRNA concentration.
(MOV)

Movie S3 **A 7×3 slab of growing PSM started with 100% noise added, without active Delta-Notch cis-inhibition.** Shown is the cytoplasmic *Hes7* mRNA concentration.
(MOV)

Movie S4 **A 7×3 slab of growing PSM started with 100% noise added, with active Delta-Notch cis-inhibition.** Shown is the cytoplasmic *Hes7* mRNA concentration.
(MOV)

Movie S5 **A 7×3 slab of growing PSM with a 20 minute shut-down of transcription in mitotic cells (red) and active Delta-Notch cis-inhibition.** Shown is the cytoplasmic *Hes7* mRNA concentration.
(MOV)

Movie S6 **A 7×3 slab of growing PSM with a 20 minute shut-down of transcription in mitotic cells (red) but without active Delta-Notch cis-inhibition.** Shown is the cytoplasmic *Hes7* mRNA concentration.
(MOV)

Text S1 **Parameter discussion for a 2-cell system.** The R measure for synchronization is discussed and shown for the variation of parameters for a 2-cell system with and without D/N cis-inhibition for three different initial conditions.
(DOC)

Text S2 **Download information including a mini manual of the program.** The file ‘HowToInstallAndRunTheProgram’ explains how to install the program on different operating systems and how to use the graphical user interface.
(PDF)

Text S3 **SBML file for the model described in** **Figure 1** **.** The file ‘SBML_DeltaNotchModel_2cells_cis.xml’ includes a SBML-description of our model described in Fig. 1.
(XML)

Text S4 **SBML file for the model described in Figure S1.** The file ‘SBML_DeltaNotchModel_2cells_cis.xml’ includes a SBML-description of the model with LFNG modulation of D/N-signaling included as described in Fig. S1.
(XML)
