# Supplementary figures and images for: Fast Synchronization of Ultradian Oscillators Controlled by Delta-Notch Signaling with Cis-Inhibition
Source: PLoS Comput Biol. 2014 Oct 2;10(10):e1003843. doi: 10.1371/journal.pcbi.1003843 (PMC4196275; doi:10.1371/journal.pcbi.1003843)

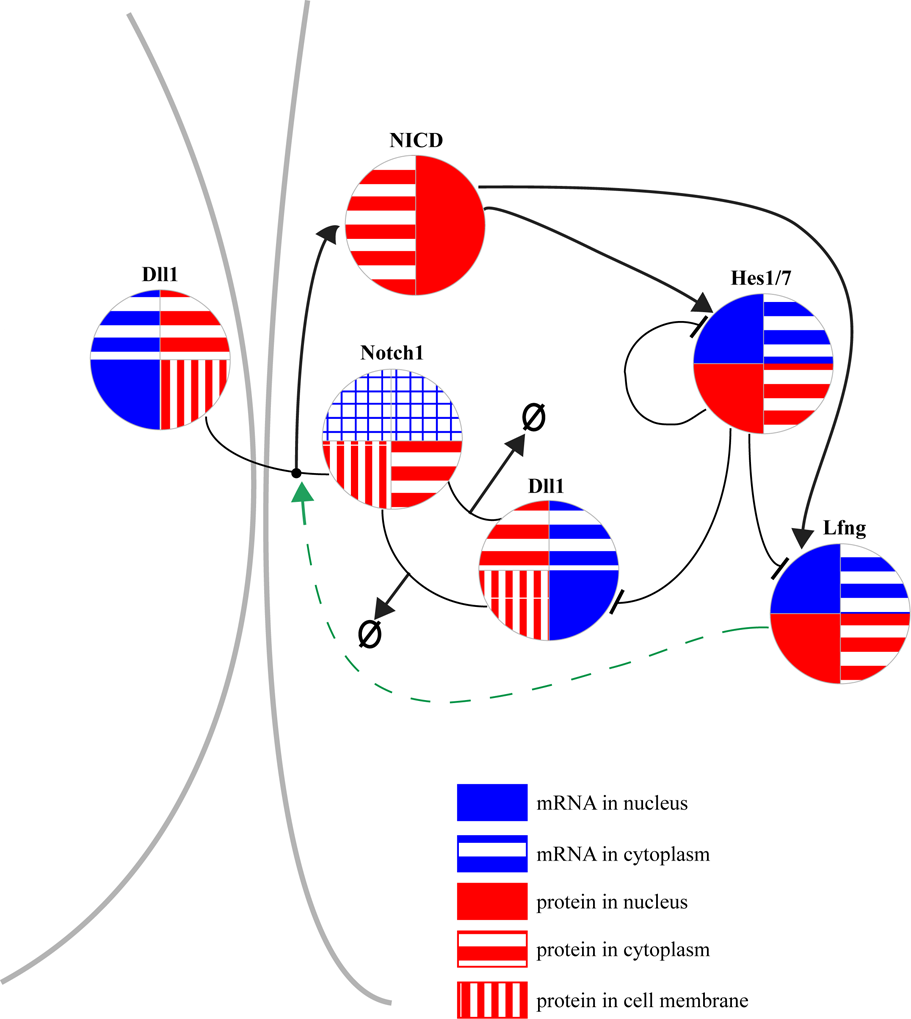

Supplement: Figure S1 — The gene regulatory network with the gene Lfng included. Color codes like in Fig. 1. (TIF) [file pcbi.1003843.s001.tif]

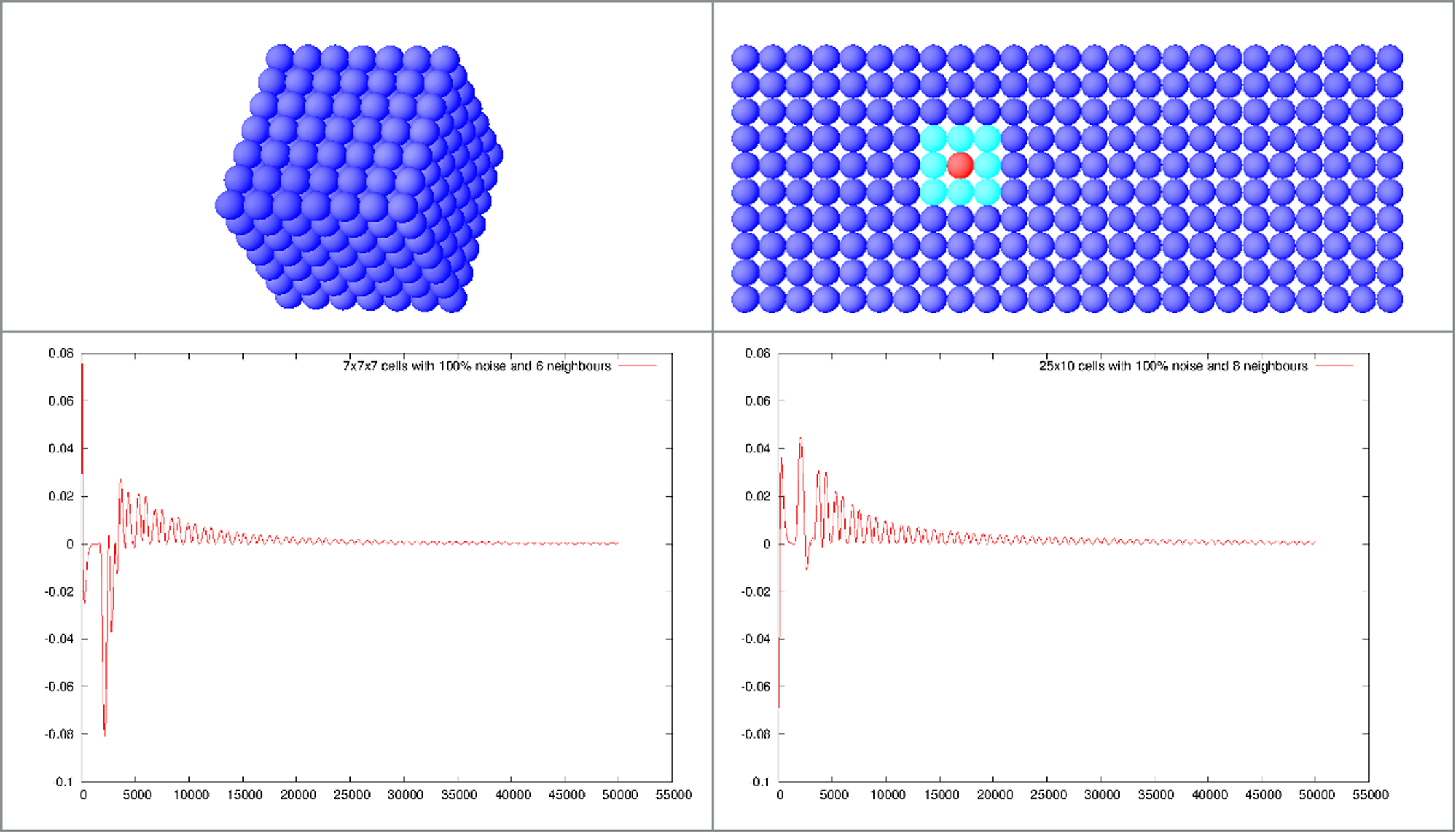

Supplement: Figure S2 — Virtual expression patterns for Hes7 mRNA for systems of different dimensionality. Virtual expression patterns for Hes7 mRNA (top row) at time point 680 min for systems of different dimensionality. At the bottom the time course of our correlation function is displayed, which shows how the different systems approach the synchronized state (Correlation function = 0). At the left side a 3-dimensional system with 6 neighboring cells, at the right a 2-dimensional system with 8 neighboring cells (light blue) signaling to the red cell. (100% ‘noise’ added). (TIF) [file pcbi.1003843.s002.tif]

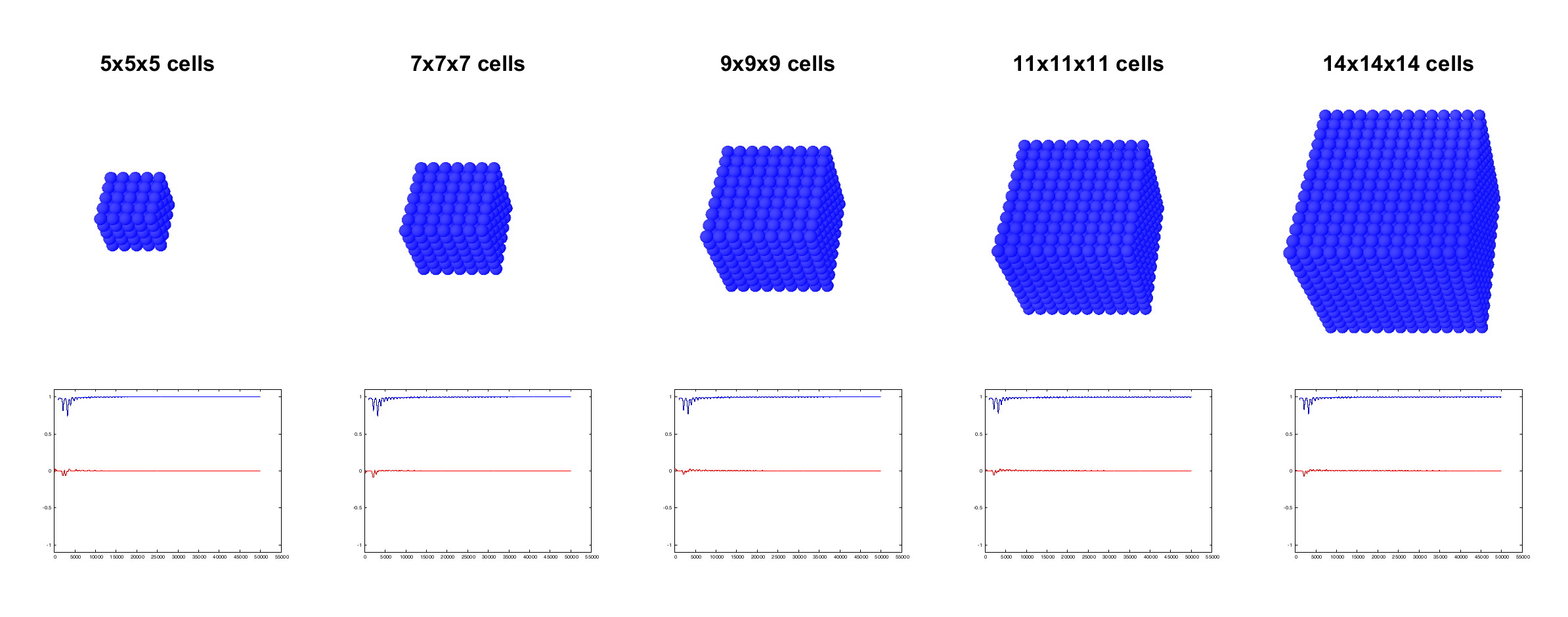

Supplement: Figure S3 — Time course of synchronization for different volumes. In the bottom panel time courses of correlation functions C(t) (red curve) and synchronization measures R (blue curve) for systems of different volumes (with 100% noise added) are shown. The top panel shows Hes7 mRNA expression in the cubes with edge lengths of 5, 7, 9, 11, and 14 cells, respectively, at the end of each simulation run. (TIF) [file pcbi.1003843.s003.tif]

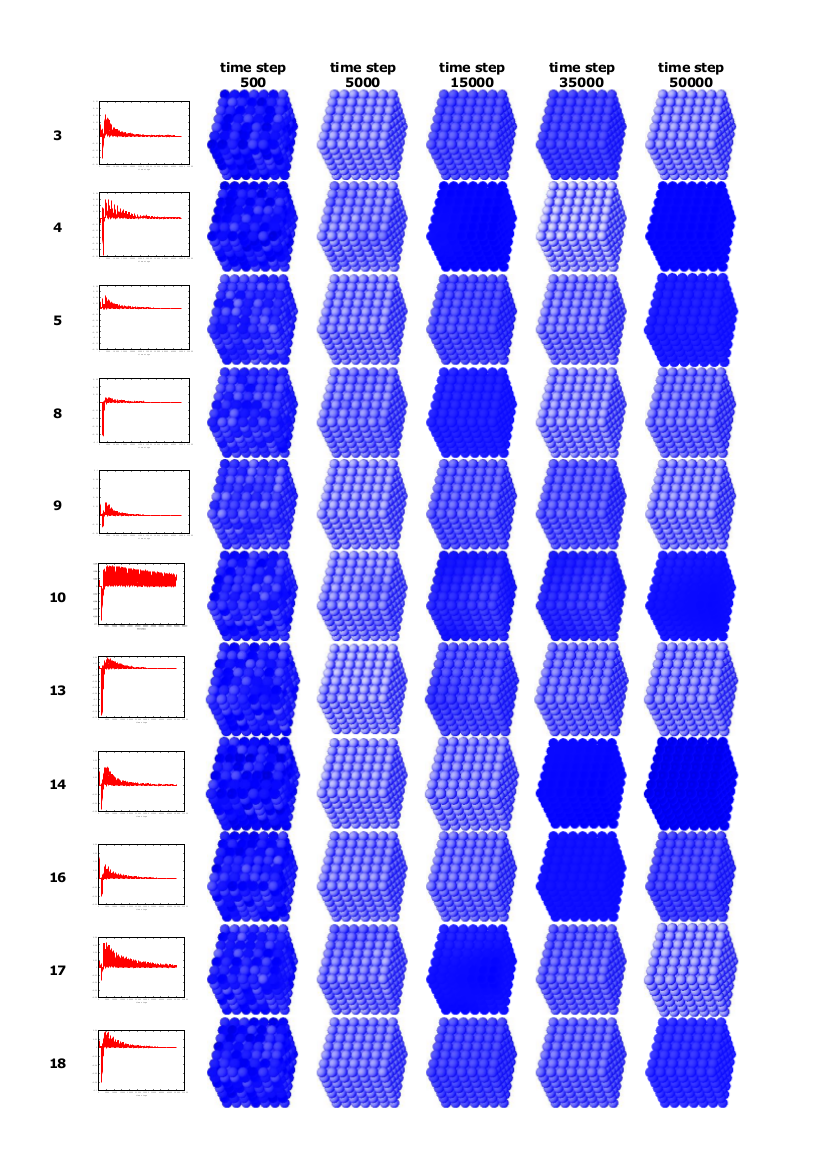

Supplement: Figure S4 — Virtual expression patterns for Hes7 mRNA resulting from different parameter sets. Snapshots of Hes7 mRNA at 500, 5000, 15000, 35000, 50000 time steps (1 time step = 0.1 min) after simulation start for a 7×7×7 cell cube for different parameter sets generate by randomly changing all transport-, production-, and decay-rates within boundaries of plus-minus ten percent of our default values. In all cases 100% noise was added at the start of the simulation. On the left side the time course of the correlation function C(t) (red curve) is shown. Parameter sets #10, #20 and #24 s result in expression patterns which don't synchronize, but show wave like behavior. (TIF) [file pcbi.1003843.s004.tif]

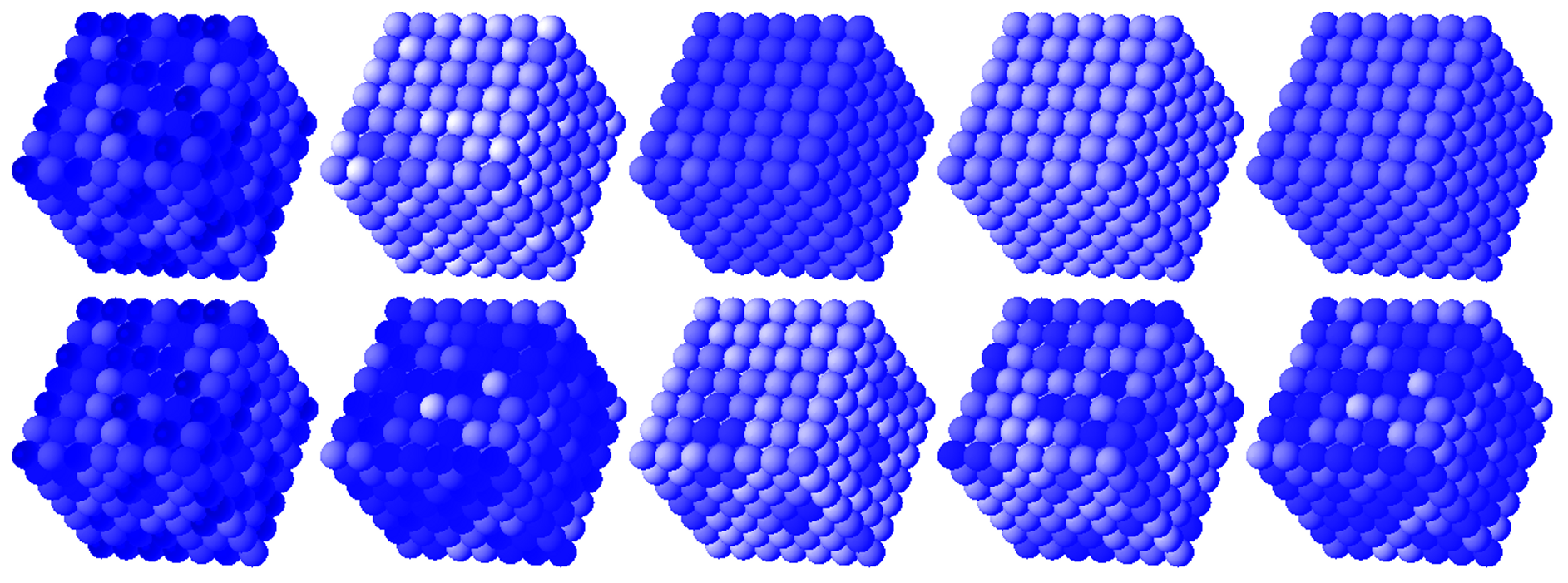

Supplement: Figure S5 — Simulation with the GRN shown in Figure S2 in each cell. Virtual expression patterns for Hes7 mRNA at 39, 220, 440, 520, 720 min after simulation start for a 7×7×7 cell cube. Top row with D/N cis-inhibition, bottom row without D/N cis-inhibition. Both cases with 150% noise added. (TIF) [file pcbi.1003843.s005.tif]
