# Supplementary material for: Fast Synchronization of Ultradian Oscillators Controlled by Delta-Notch Signaling with Cis-Inhibition
Source: PLoS Comput Biol. 2014 Oct 2;10(10):e1003843. doi: 10.1371/journal.pcbi.1003843 (PMC4196275; doi:10.1371/journal.pcbi.1003843)
Supplement: Text S1 — Parameter discussion for a 2-cell system. The R measure for synchronization is discussed and shown for the variation of parameters for a 2-cell system with and without D/N cis-inhibition for three different initial conditions. (DOC) [file pcbi.1003843.s013.doc]

# Sensitivity Analysis

In order to analyze how sensitive synchronization depends on parameter changes and initial conditions, we performed a qualitative sensitivity analysis. For this purpose, in contrast to the model discussed in the main text, we considered only a two-cell system. Furthermore, a measure of synchronization was used which takes into account also variability over time.

# Method

To characterize quantitatively synchronization in a two-cell system we use an order parameter like in [Garcia-Ojalvo:04]. The synchronization measure R is defined as the ratio of the standard deviation of the time series of the average amount of gene product to the standard deviation of gene product averaged over the number of cells,

R = Var_t_(**c**) / Mean_i_(Var_t_(c_i_))

where c_i_ is the amount of pC_h7, i=1,2, and **c**=(c_1_+c_2_)/2. To make the order parameter R less sensitive to short unsynchronized periods we used a time window of 1000 time units. Note that in contrast to the synchronization measure in the main text, above measure is 0 if no synchronization and 1 if perfect synchronization occurs.

To analyze the dependency of the order parameter R on model parameters and initial conditions of the two-cell system, we varied each parameter by +/- 20% around the default value while keeping all other parameters fixed. The default parameter values are displayed in the middle column of Table 1. In the system with cis-inhibition the two parameters r_DNcisC and r_DNcisM were set equal to 0.01. A summary of the parameter ranges is listed in the third column of Table 1. The dependency on initial conditions was performed for three different state configurations, listed in Table 2. Note that the initial values of the first cell are the same in all three configurations. Only the initial values of the second cell were varied.

The speed of synchronization was analyzed for the three sets of initial conditions mentioned above. Furthermore, each parameter was drawn randomly from a uniform distribution on an interval of +/- 20% from its default value while keeping all other parameters fixed. The time dependency of the synchronization measure was calculated by shifting a time window of 1000 time units, starting at zero, by 5 time units per step along the positive time axis.

# Results

## Parameter-dependent synchronization

Sensitivity analysis is performed for each parameter on an interval of +/- 20% around the standard values for three different initial conditions.

Table 1 Default parameter values and sensitivity range of model parameters in a two-cell system.

| **Parameter** | **Default Value** | **Sensitivity Range** |
| --- | --- | --- |
| HCoef_nic | 2.3 | (1.84,2.76) |
| HCon_nic | 6 | (4.8,7.2) |
| K | 1.5 | (1.2,1.8) |
| dpC_h7 | 0.031 | (0.0248,0.0372) |
| epC_h7 | 0.007 | (0.0056,0.0084) |
| epN_h7 | 0.001 | (8e-04,0.0012) |
| G_h7 | 0.96 | (0.768,1.152) |
| F_h7 | 0.2 | (0.16,0.24) |
| emN_h7 | 0.038 | (0.0304,0.0456) |
| dmC_h7 | 0.067 | (0.0536,0.0804) |
| k_h7 | 0.5 | (0.4,0.6) |
| dmN_h7 | 0.001 | (8e-04,0.0012) |
| r_DN | 0.05 | (0.04,0.06) |
| dpC_nic | 0.2 | (0.16,0.24) |
| epC_nic | 0.12 | (0.16,0.24) |
| epN_nic | 0.06 | (0.048,0.072) |
| epC_nic | 0.12 | (0.16,0.24) |
| G_nic | 0.02 | (0.016,0.024) |
| F_nic | 5 | (4,6) |
| epN_nic | 0.06 | (0.048,0.072) |
| K_d1 | 1.5 | (1.2,1.8) |
| dpC_d1 | 0.09 | (0.072,0.108) |
| epC_d1 | 0.1 | (0.08,0.12) |
| epM_d1 | 0.1 | (0.08,0.12) |
| emN_d1 | 0.09 | (0.072,0.108) |
| dmC_d1 | 0.12 | (0.096,0.144) |
| dmN_d1 | 0.001 | (8e-04,0.0012) |
| kk_d1 | 1.25 | (1,1.5) |
| K_n1 | 1.5 | (1.2,1.8) |
| dpC_n1 | 0.2 | (0.16,0.24) |
| epC_n1 | 0.1 | (0.08,0.12) |
| dpM_n1 | 0.1 | (0.08,0.12) |
| kk_n1 | 0.5 | (0.4,0.6) |
| dm_n1 | 0.02 | (0.016,0.024) |

Table 2 Initial conditions of the two-cells system

| Initial1 | Initial2 | Initial3 |
| --- | --- | --- |
| Sensitivity analysis with initial conditions 1 | Sensitivity analysis with initial conditions 2 | Sensitivity analysis with initial conditions 3 |
| |  | **state** | | --- | --- | | pC_h7_1 | 105 | | pN_h7_1 | 0.5 | | mC_h7_1 | 4.4 | | mN_h7_1 | 12.4 | | pC_nic_1 | 5 | | pN_nic_1 | 5 | | pC_d1_1 | 5 | | pM_d1_1 | 5 | | mC_d1_1 | 1 | | mN_d1_1 | 5 | | pC_n1_1 | 10 | | pM_n1_1 | 5 | | m_n1_1 | 10 | | pC_h7_2 | 50 | | pN_h7_2 | 0.4 | | mC_h7_2 | 3.4 | | mN_h7_2 | 8 | | pC_nic_2 | 5 | | pN_nic_2 | 5 | | pC_d1_2 | 0.7 | | pM_d1_2 | 3 | | mC_d1_2 | 0.7 | | mN_d1_2 | 3 | | pC_n1_2 | 5 | | pM_n1_2 | 2.5 | | m_n1_2 | 5 | | |  | **state** | | --- | --- | | pC_h7_1 | 105 | | pN_h7_1 | 0.5 | | mC_h7_1 | 4.4 | | mN_h7_1 | 12.4 | | pC_nic_1 | 5 | | pN_nic_1 | 5 | | pC_d1_1 | 5 | | pM_d1_1 | 5 | | mC_d1_1 | 1 | | mN_d1_1 | 5 | | pC_n1_1 | 10 | | pM_n1_1 | 5 | | m_n1_1 | 10 | | pC_h7_2 | 120 | | pN_h7_2 | 0.4 | | mC_h7_2 | 5.4 | | mN_h7_2 | 15 | | pC_nic_2 | 2.5 | | pN_nic_2 | 7.5 | | pC_d1_2 | 5 | | pM_d1_2 | 5 | | mC_d1_2 | 1 | | mN_d1_2 | 5 | | pC_n1_2 | 15 | | pM_n1_2 | 7.5 | | m_n1_2 | 15 | | |  | **state** | | --- | --- | | pC_h7_1 | 105 | | pN_h7_1 | 0.5 | | mC_h7_1 | 4.4 | | mN_h7_1 | 12.4 | | pC_nic_1 | 5 | | pN_nic_1 | 5 | | pC_d1_1 | 5 | | pM_d1_1 | 5 | | mC_d1_1 | 1 | | mN_d1_1 | 5 | | pC_n1_1 | 10 | | pM_n1_1 | 5 | | m_n1_1 | 10 | | pC_h7_2 | 80 | | pN_h7_2 | 0.8 | | mC_h7_2 | 3 | | mN_h7_2 | 8 | | pC_nic_2 | 7.5 | | pN_nic_2 | 2.5 | | pC_d1_2 | 3 | | pM_d1_2 | 3 | | mC_d1_2 | 3 | | mN_d1_2 | 3 | | pC_n1_2 | 5 | | pM_n1_2 | 2 | | m_n1_2 | 5 | |

Figure 1

Parameter dependency of the order parameter R on model parameters for the two-cells system with (red solid line) and without (black solid line) cis-inhibition

|  | **Initial1** | **Initial2** | **Initial3** |
| --- | --- | --- | --- |
| K | 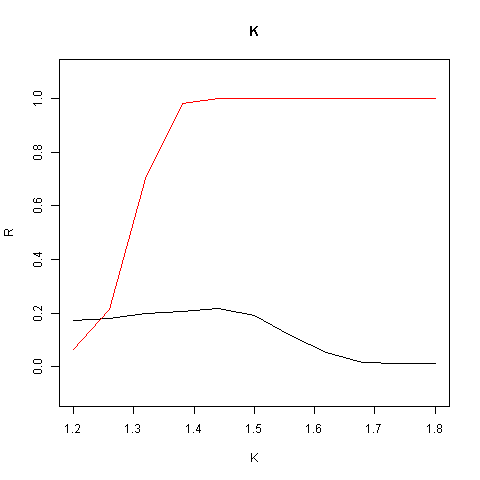 | 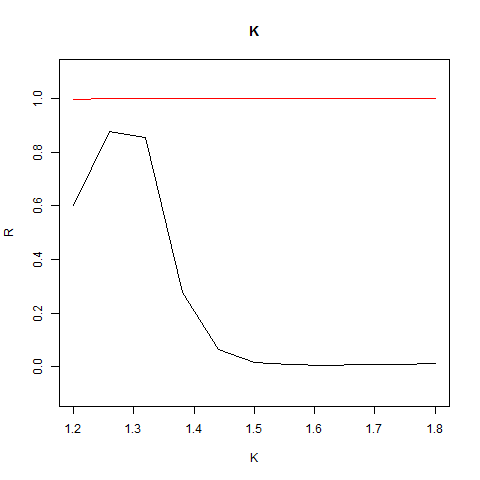 | 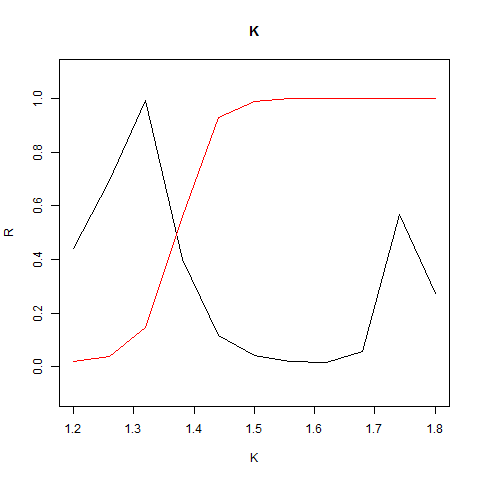 |
| dpC_h7 | 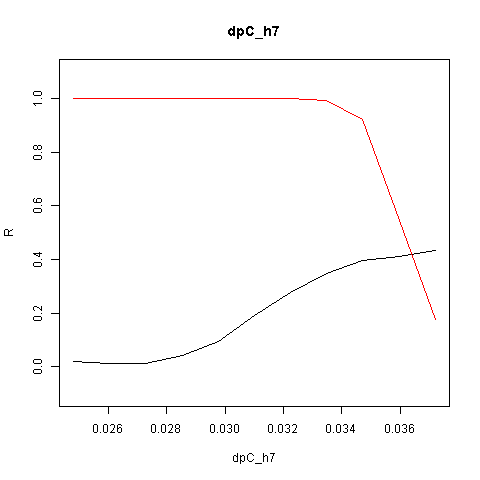 | 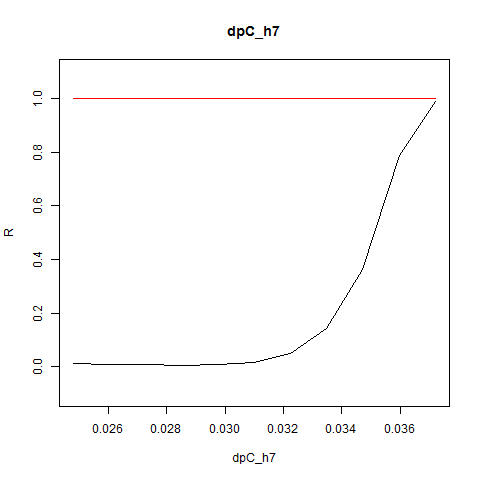 | 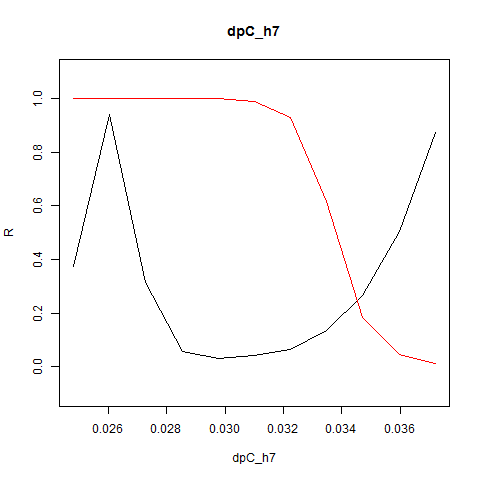 |
| G_h7 | 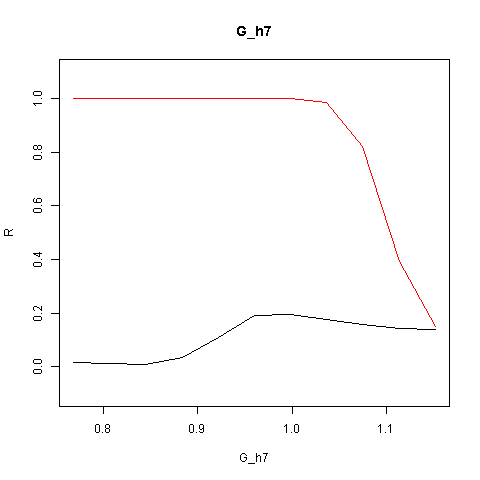 | 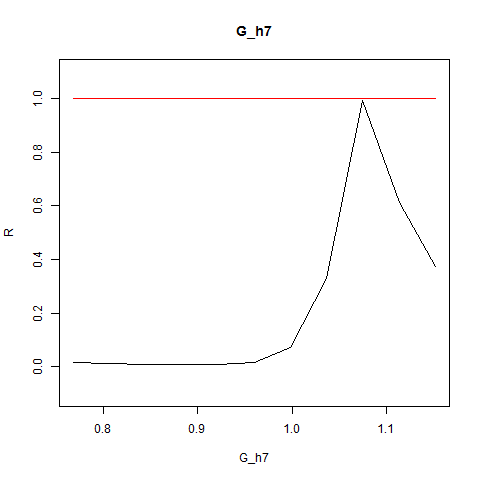 | 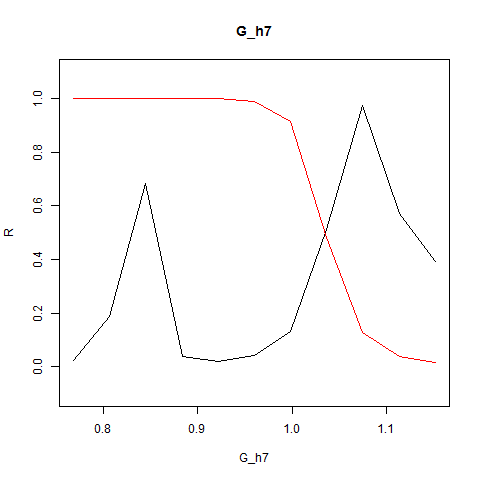 |
| F_h7 | 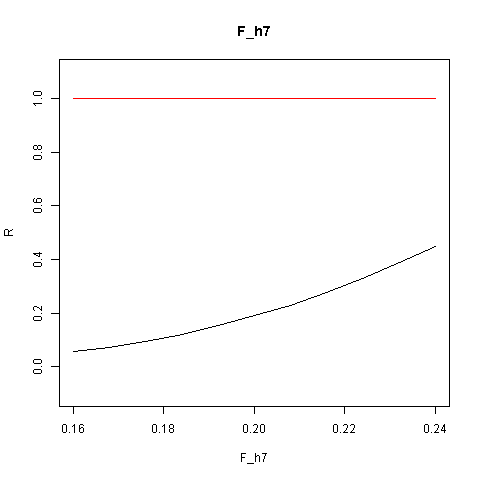 | 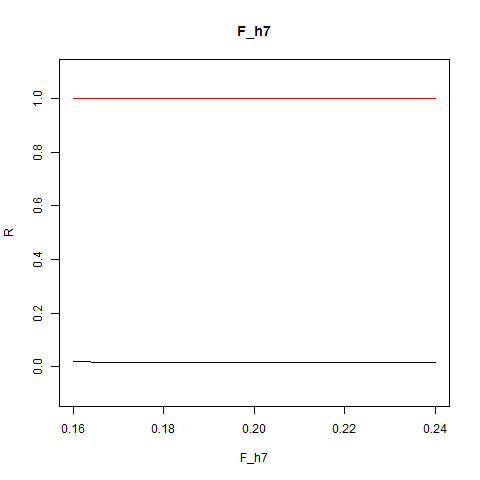 | 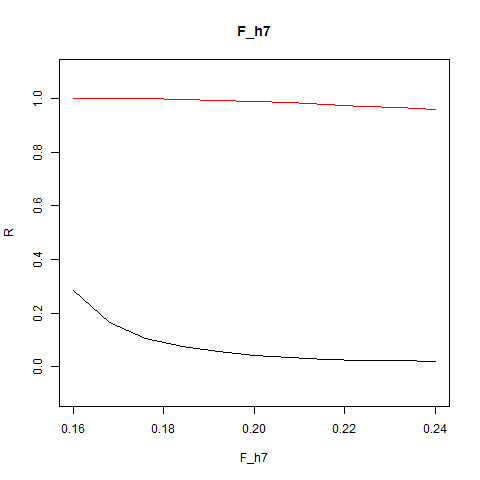 |
| epC_h7 | 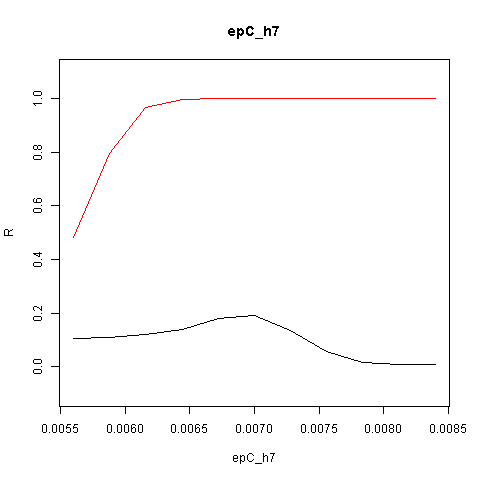 | 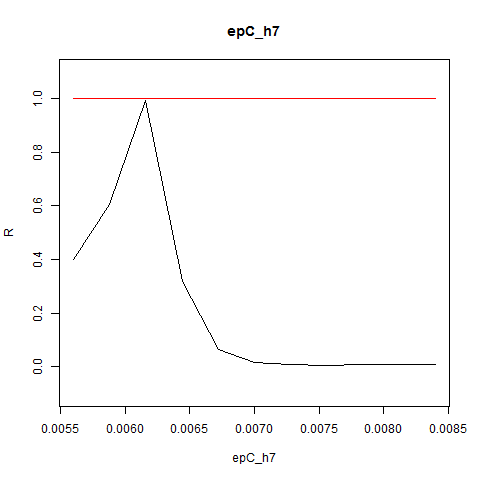 | 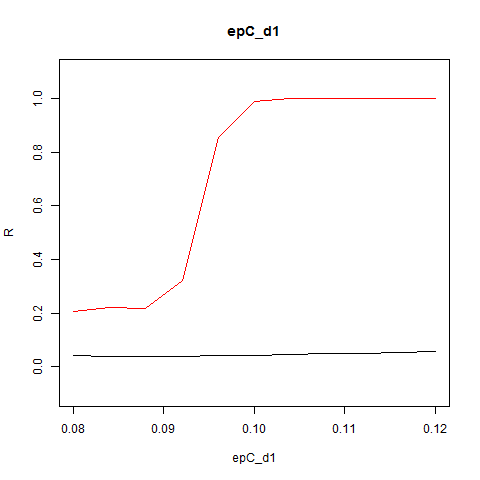 |
| epN_h7 | 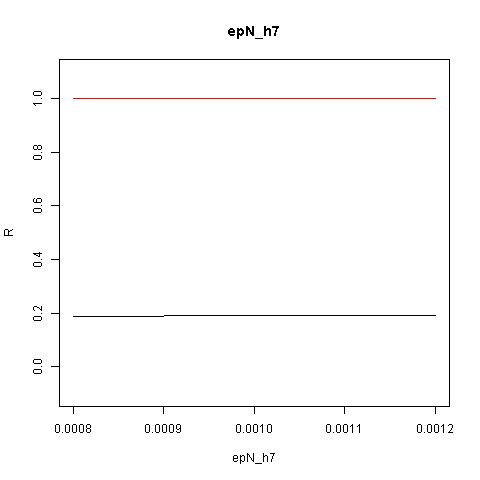 | 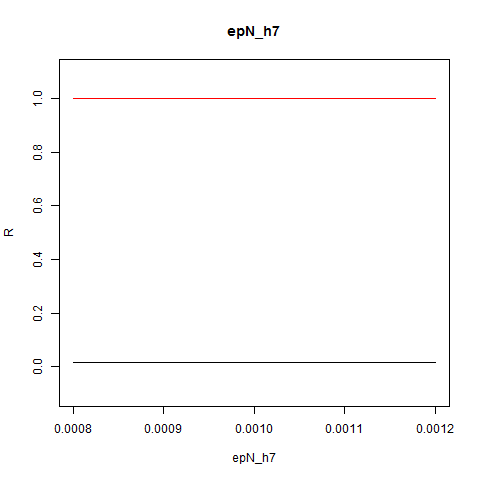 | 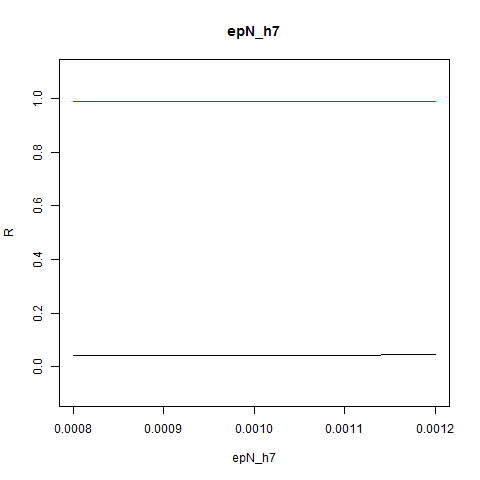 |
| k_h7 | 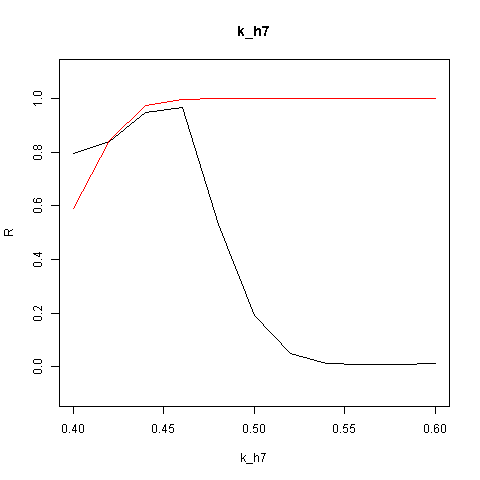 | 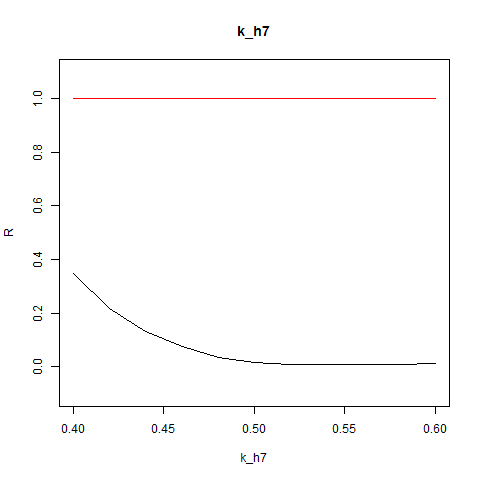 | 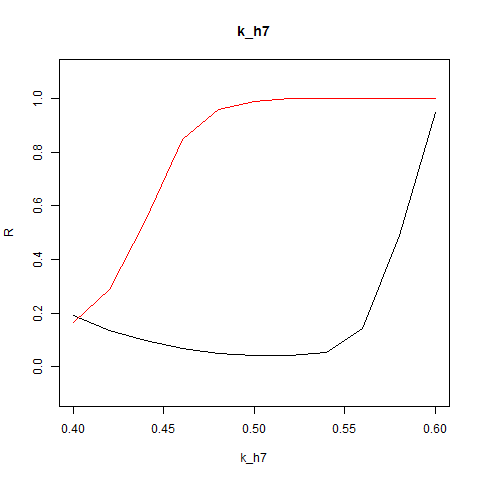 |
| dmC_h7 | 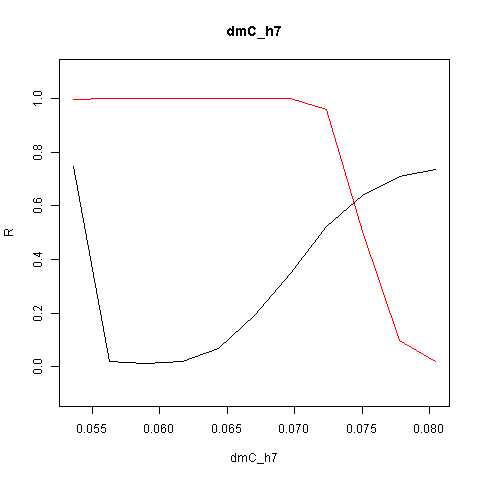 | 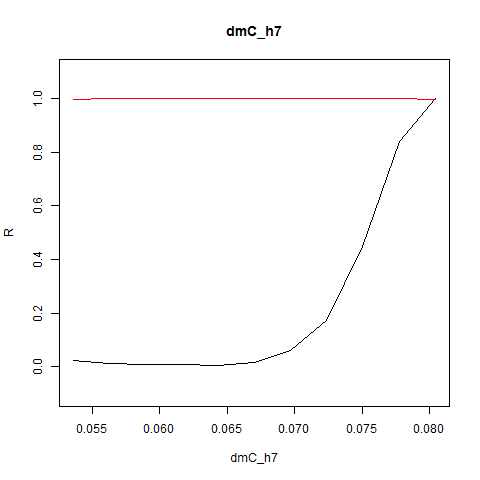 | 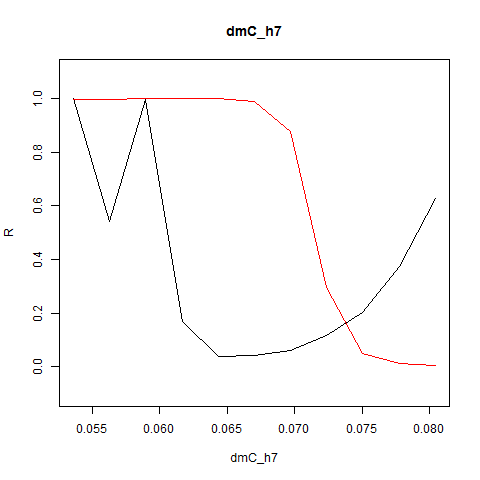 |
| dmN_h7 | 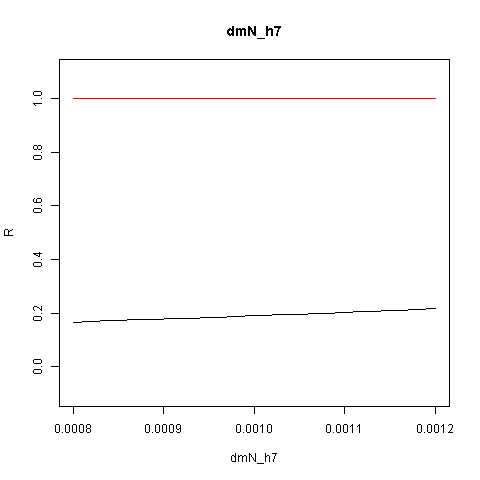 | 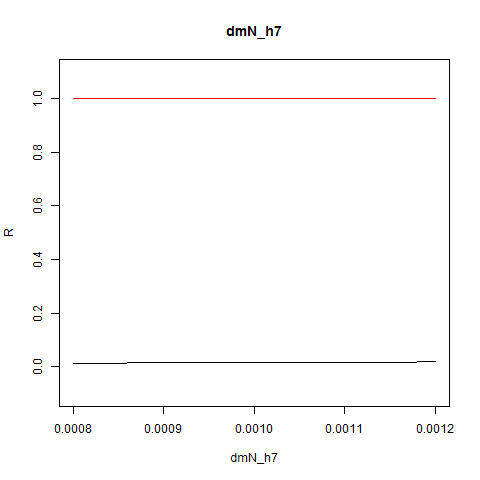 | 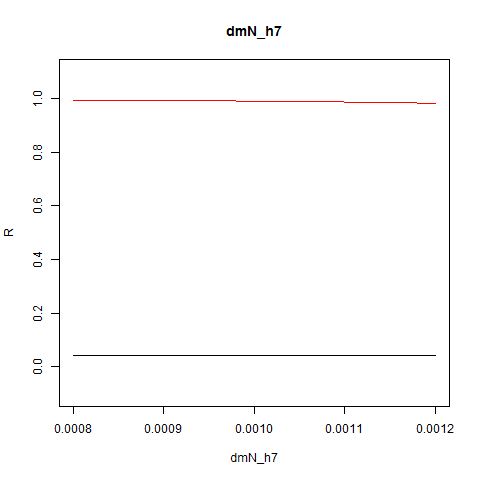 |
| emN_h7 | 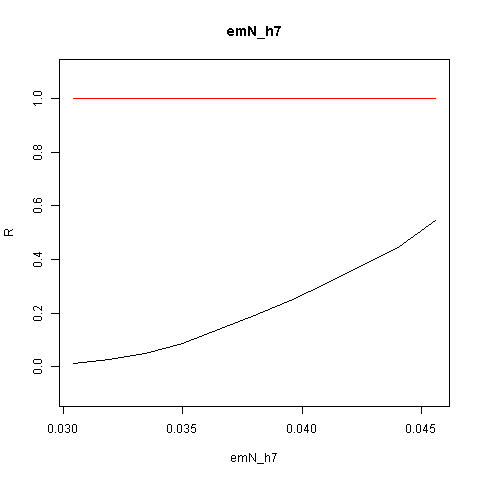 | 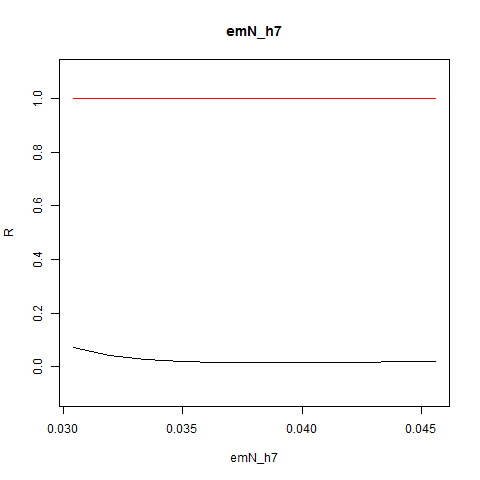 | 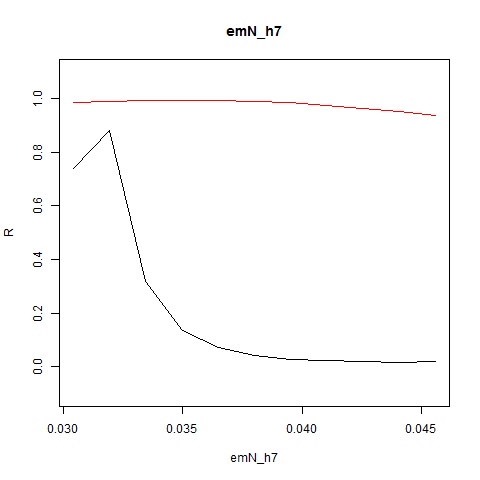 |
| HCoef_nic | 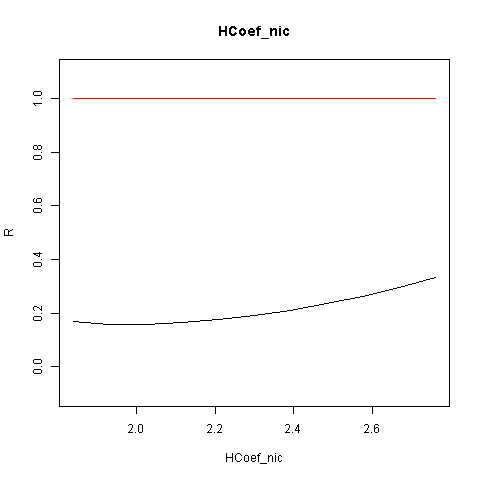 | 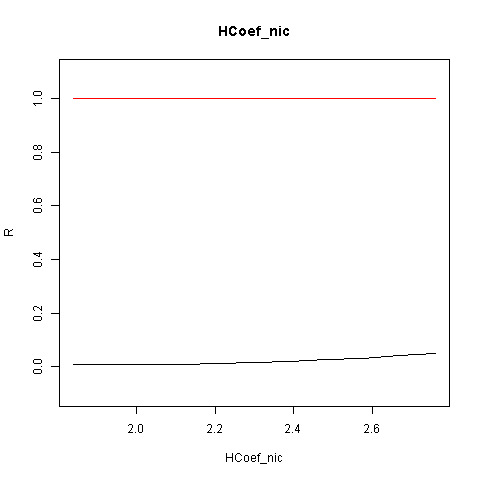 | 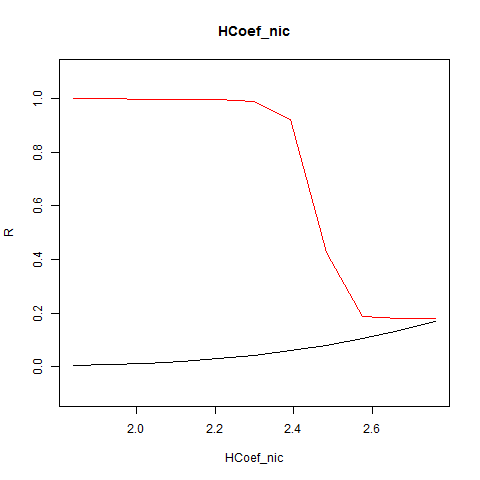 |
| Hcon_nic | 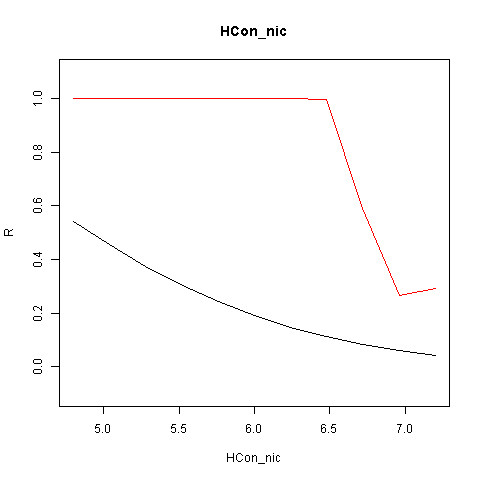 | 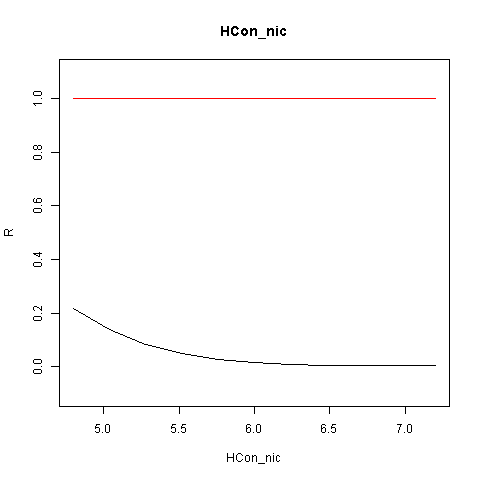 | 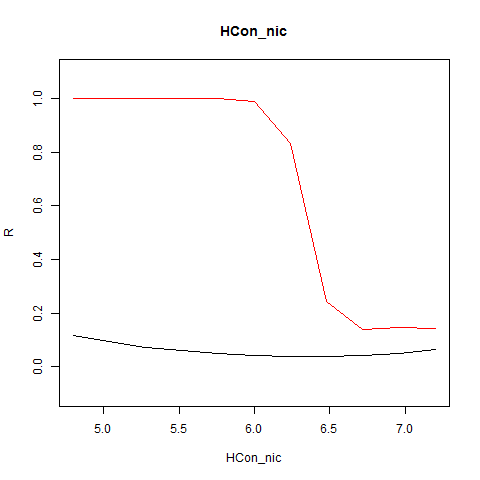 |
| r_DN | 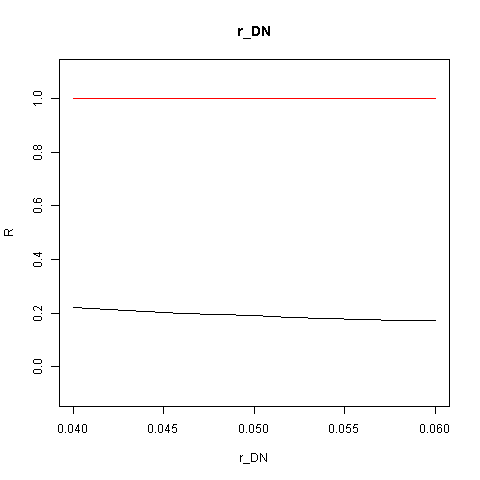 | 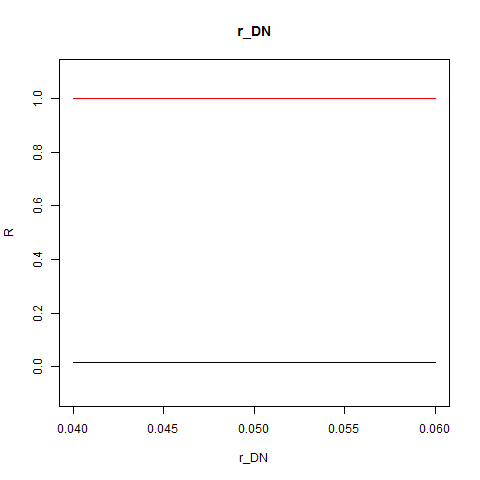 | 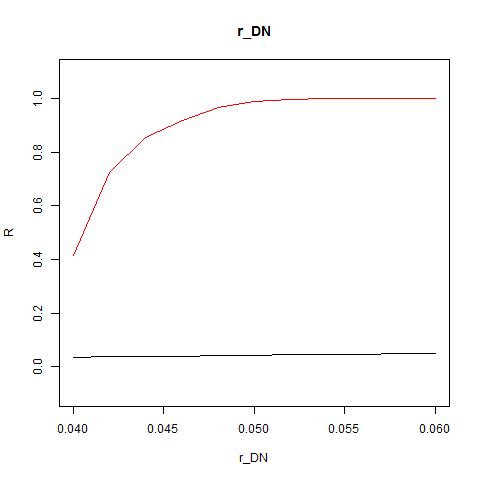 |
| r_DNcis | 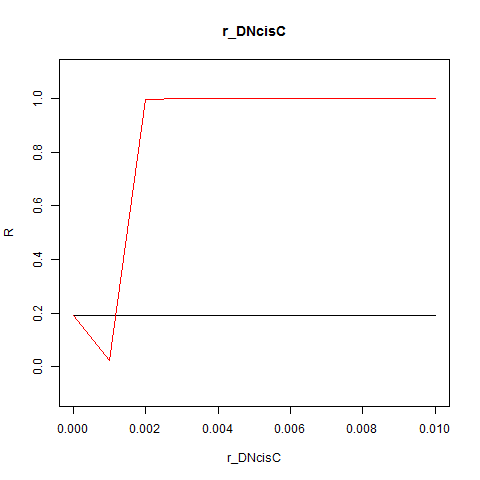 | 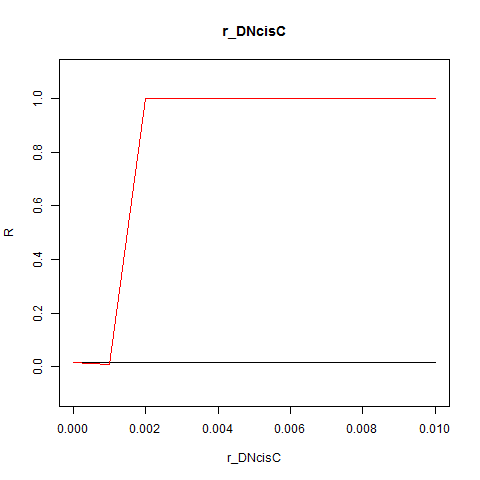 | 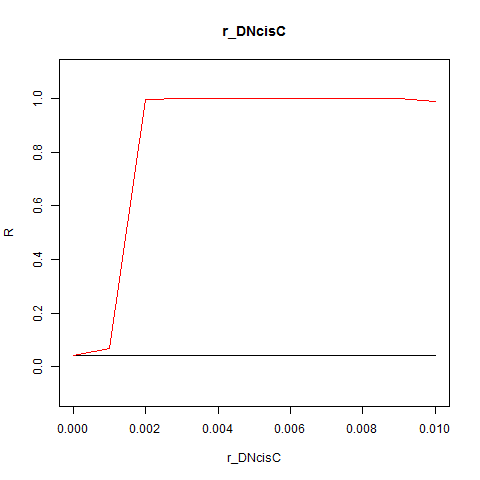 |
| dpC_nic | 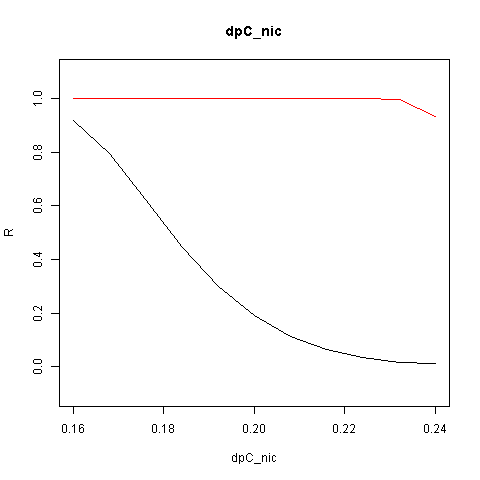 | 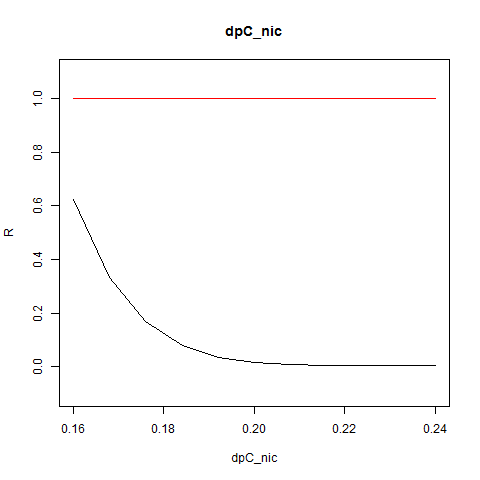 | 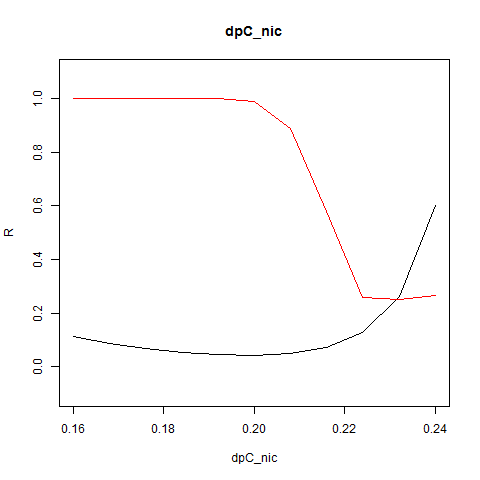 |
| G_nic | 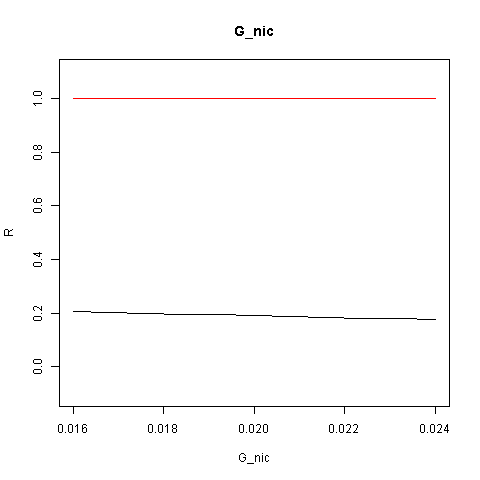 | 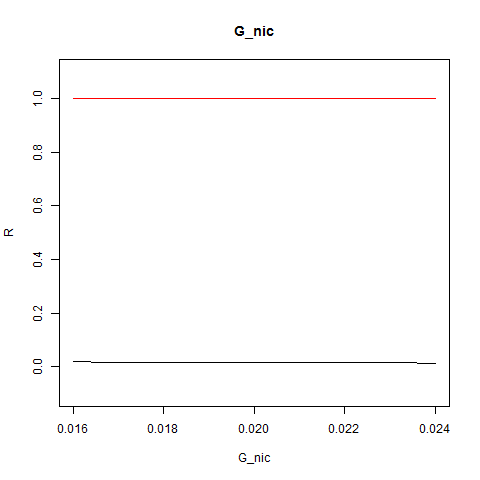 | 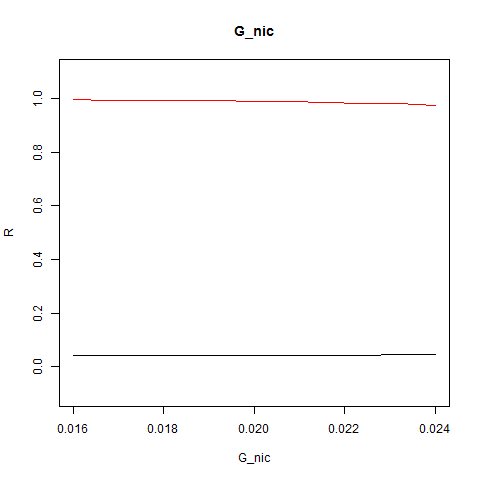 |
| F_nic | 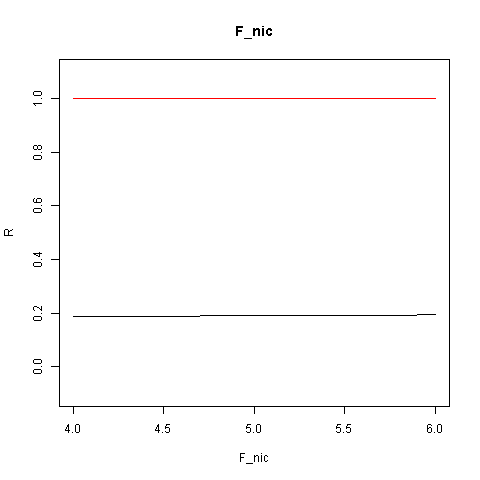 | 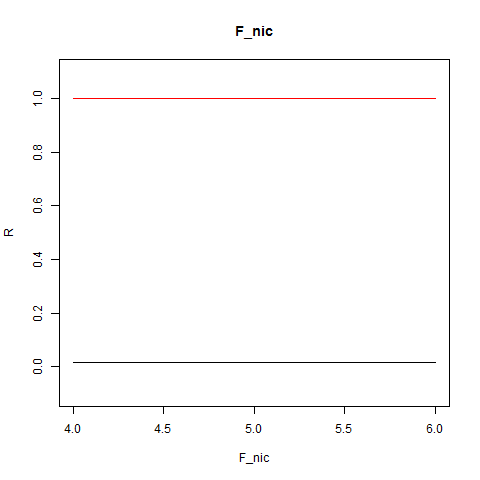 | 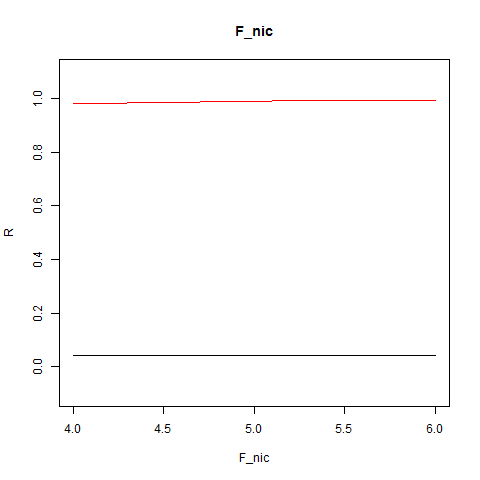 |
| epC_nic | 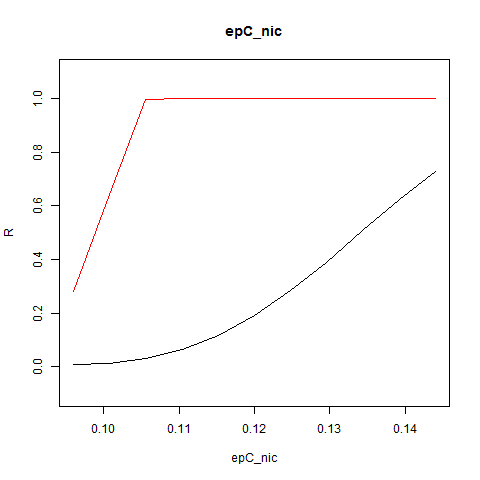 | 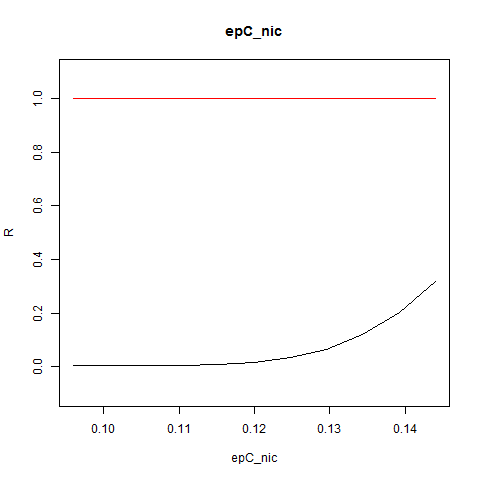 | 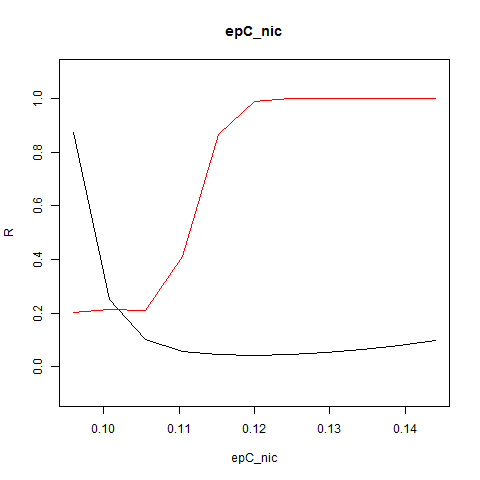 |
| epN_nic | 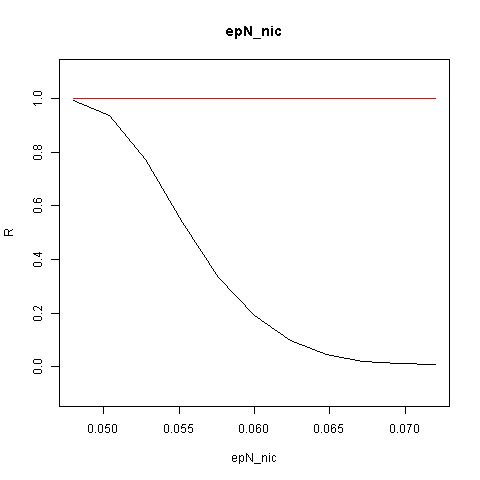 | 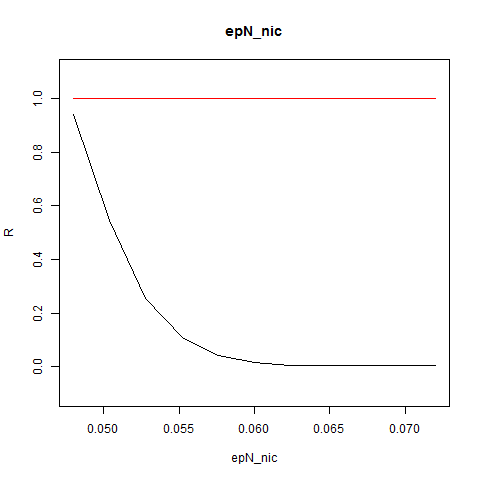 | 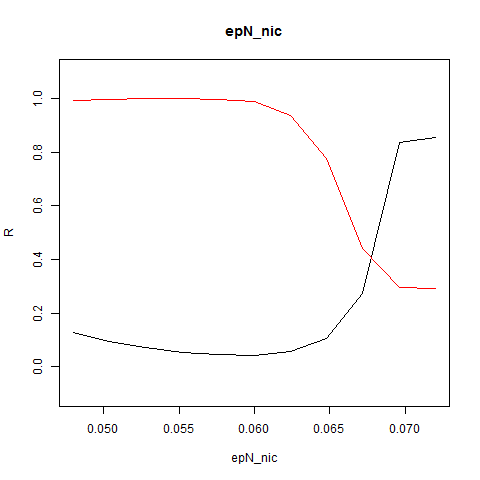 |
| K_d1 | 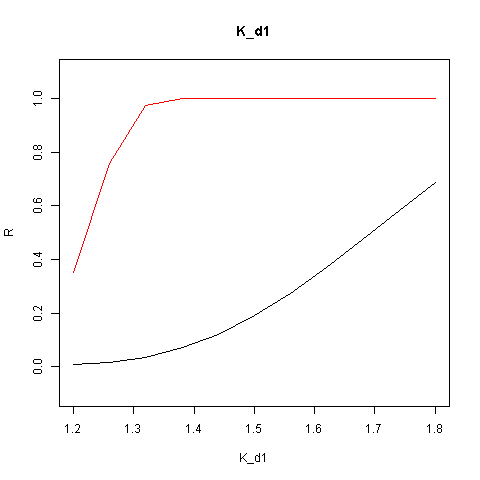 | 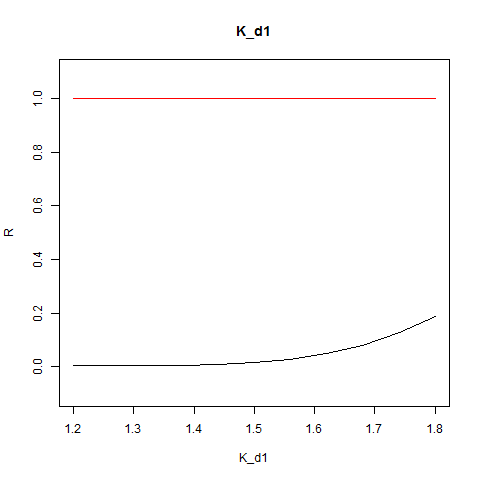 | 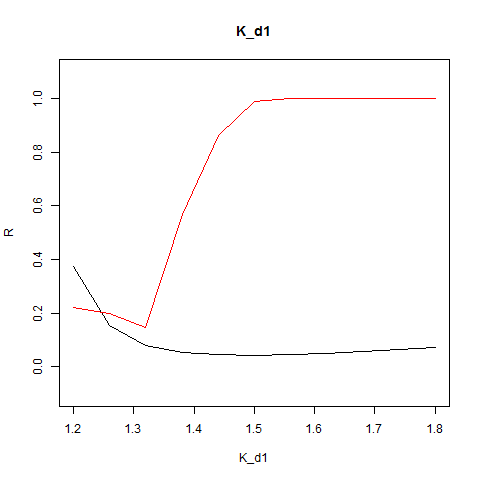 |
| dpC_d1 | 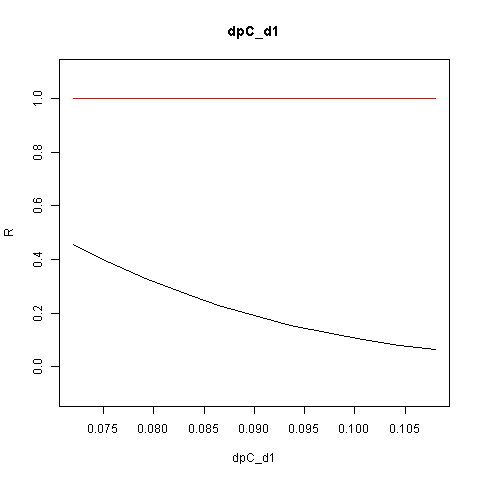 | 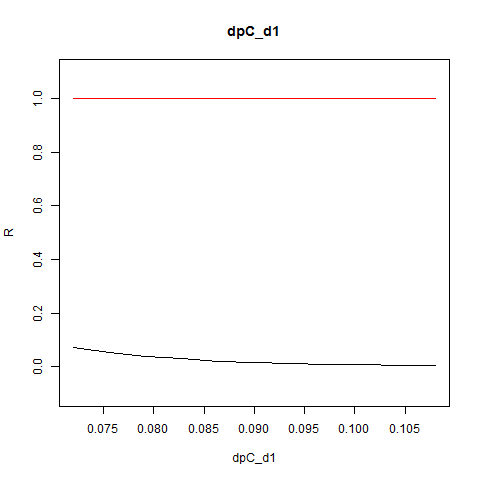 | 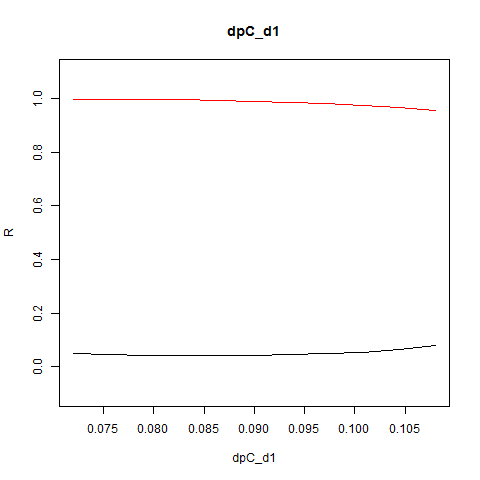 |
| epC_d1 | 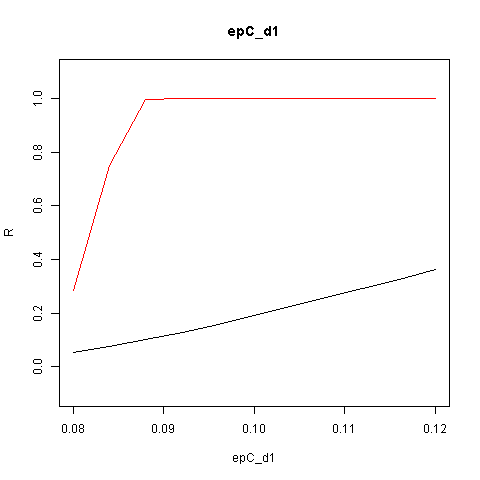 | 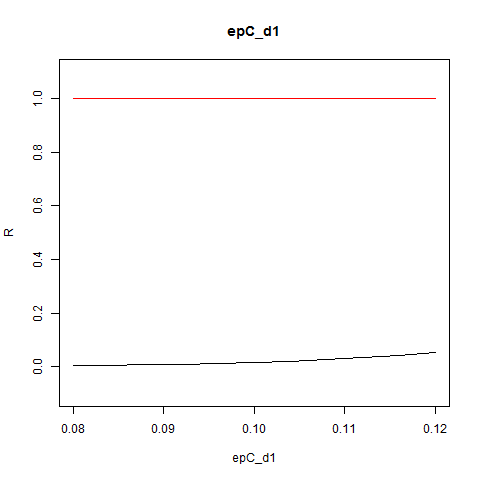 | 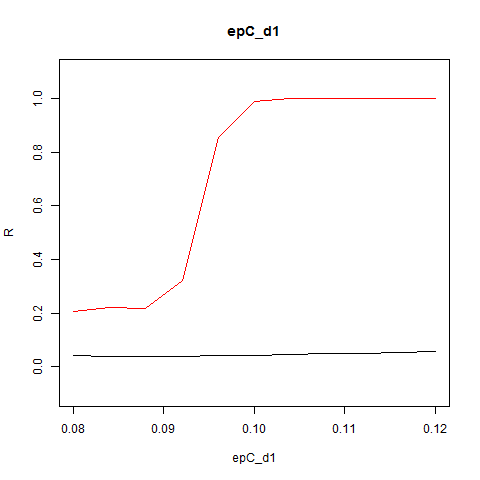 |
| epM_d1 | 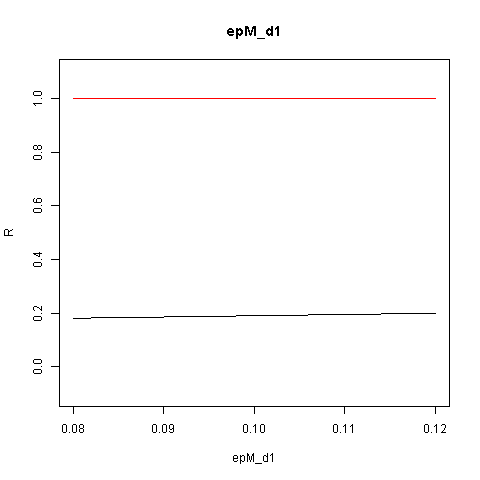 | 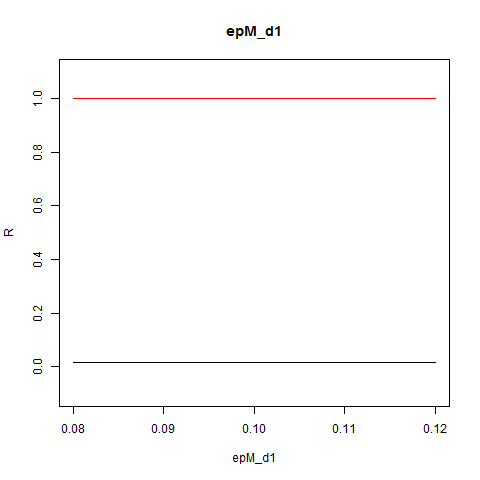 | 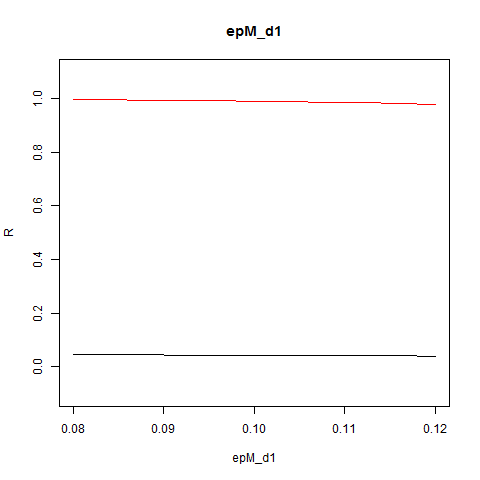 |
| dmC_d1 | 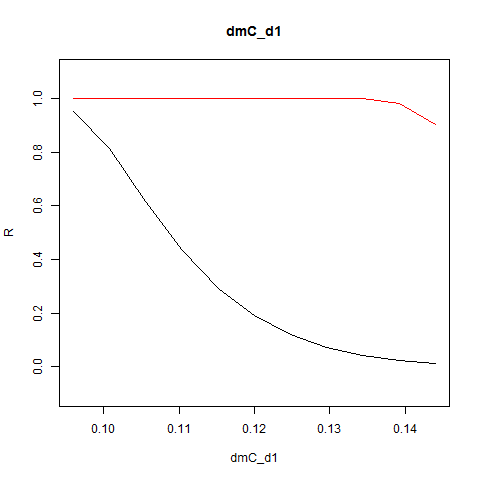 | 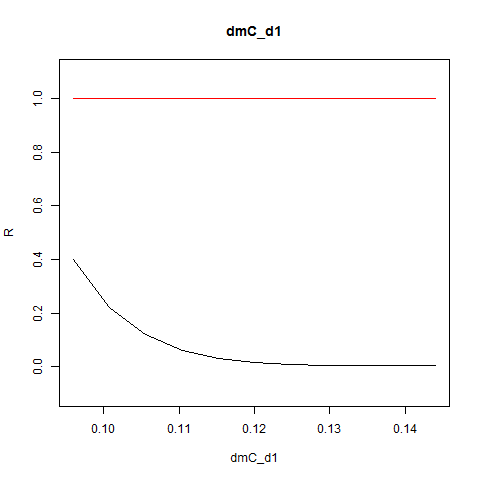 | 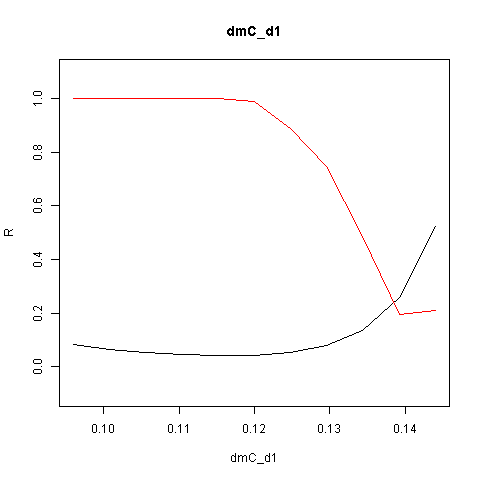 |
| dmN_d1 | 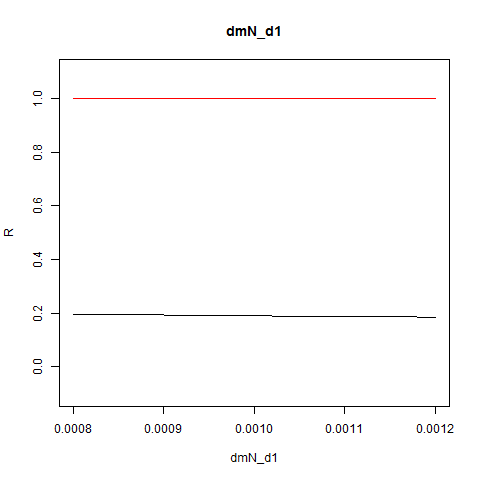 | 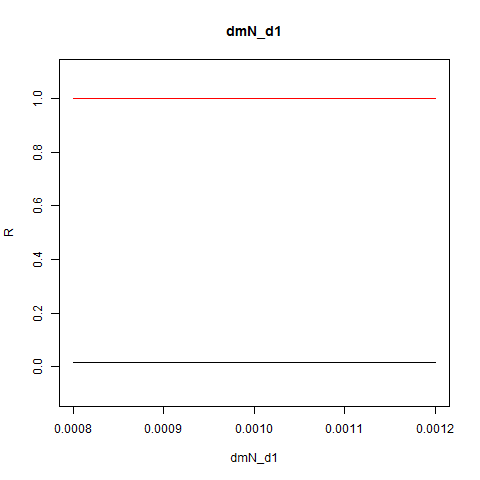 | 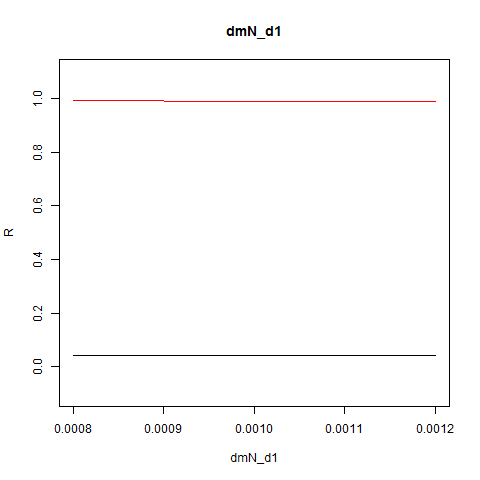 |
| emN_d1 | 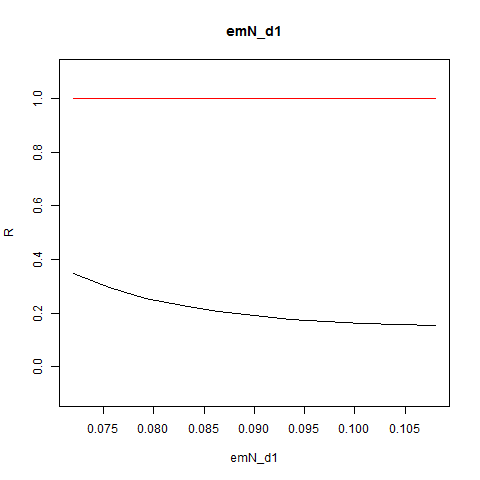 | 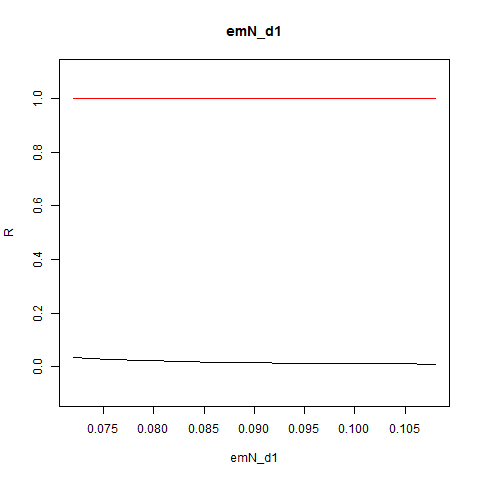 | 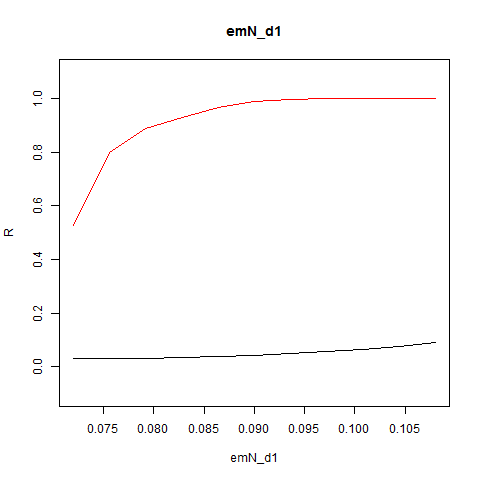 |
| kk_d1 | 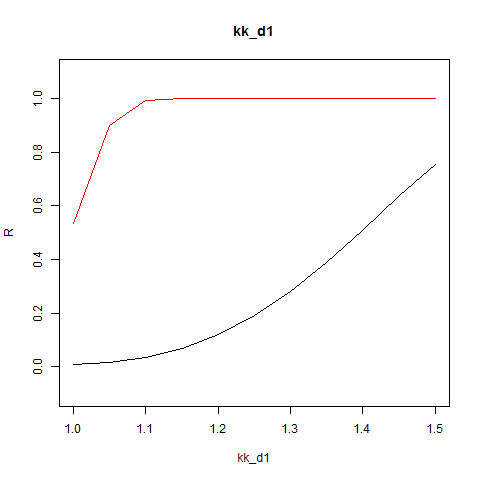 | 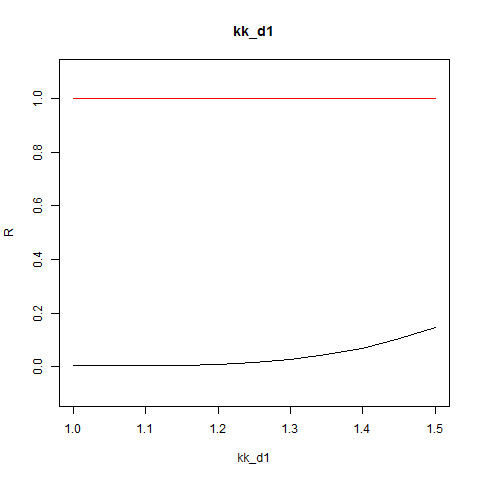 | 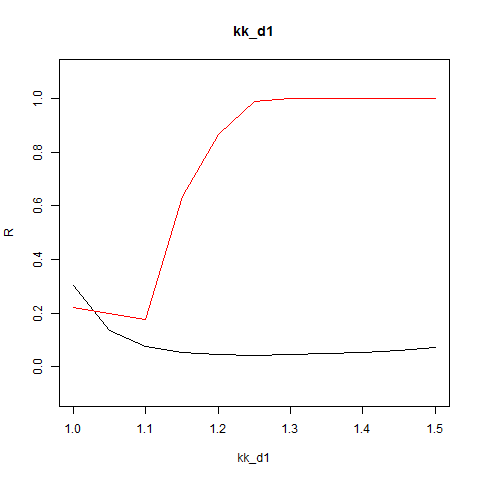 |
| K_n1 | 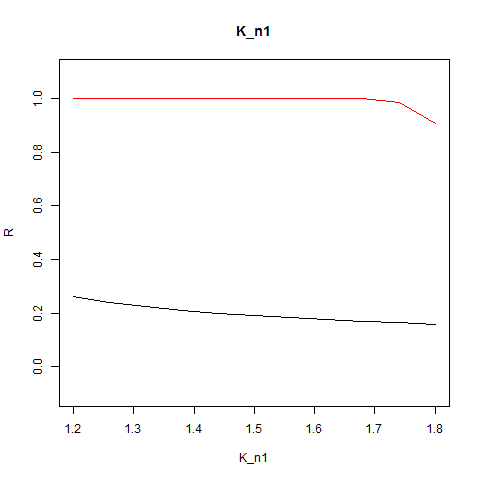 | 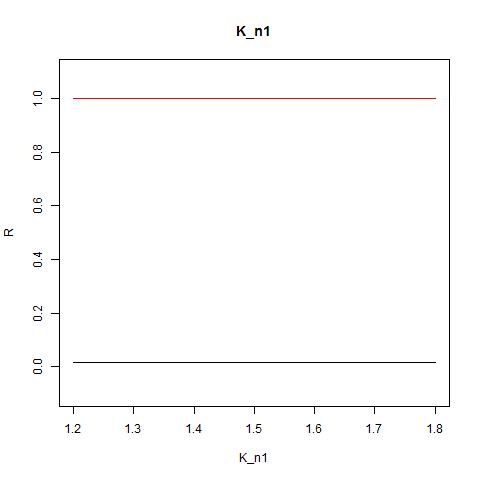 | 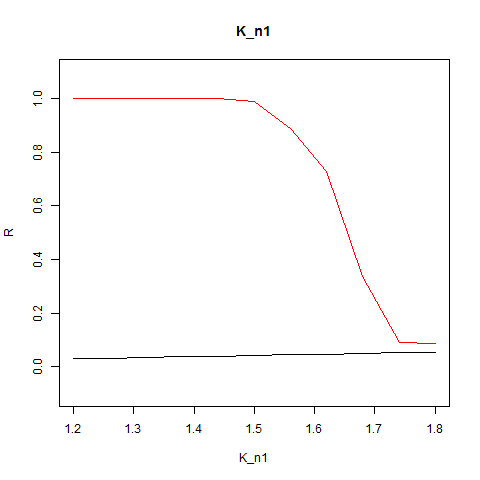 |
| dpC_n1 | 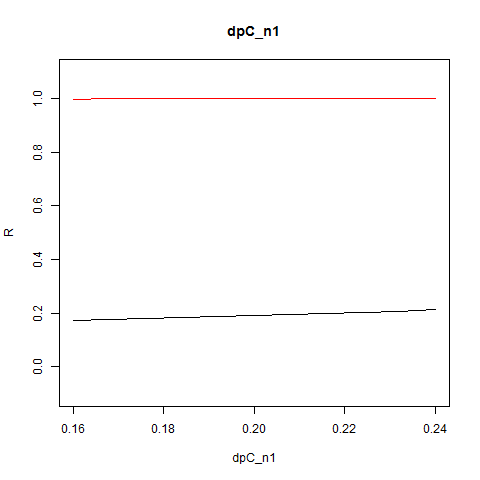 | 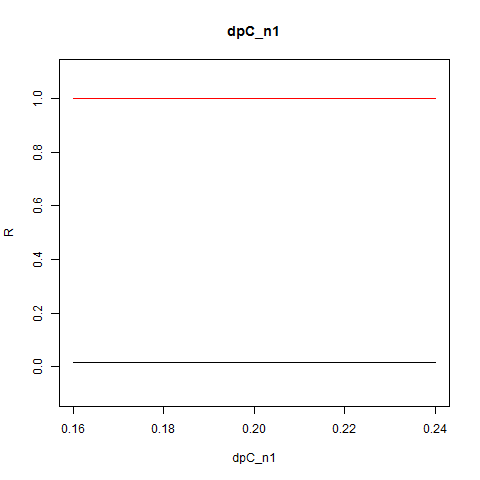 | 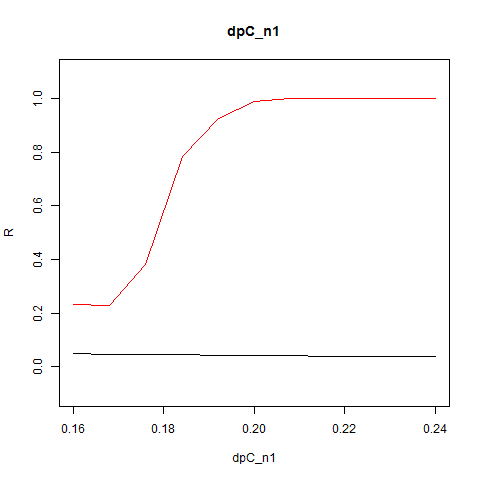 |
| epC_n1 | 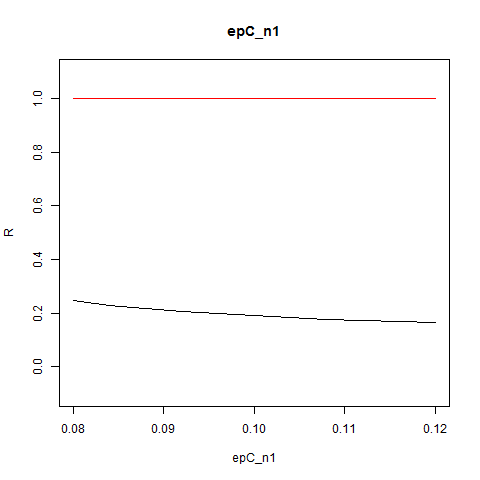 | 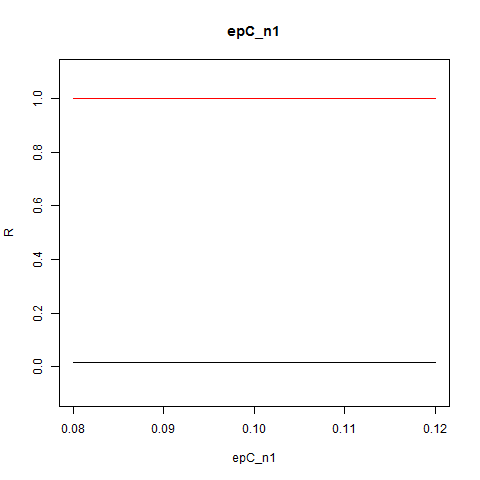 | 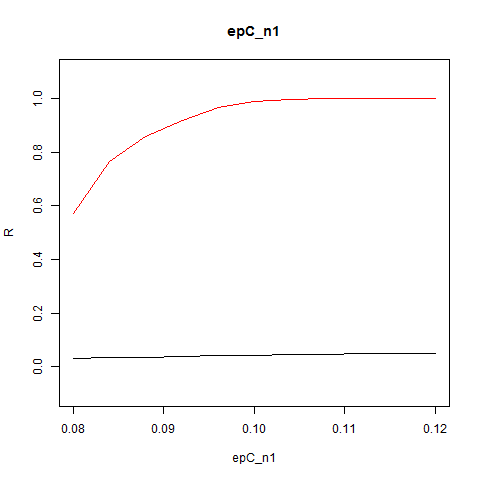 |
| dpM_n1 | 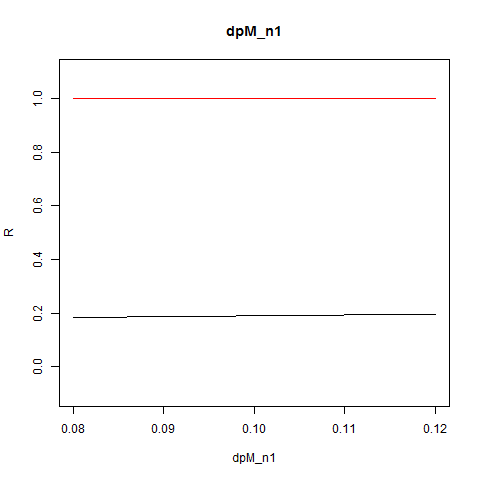 | 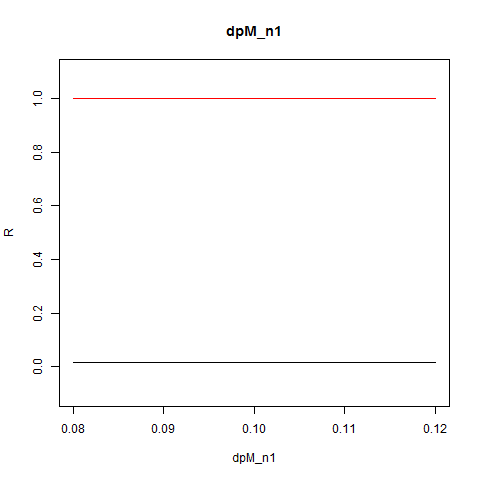 | 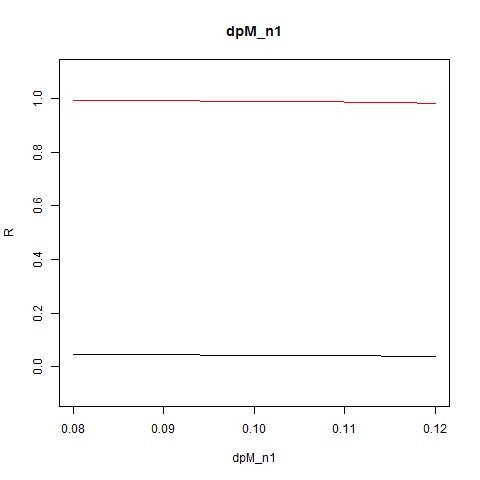 |
| kk_n1 | 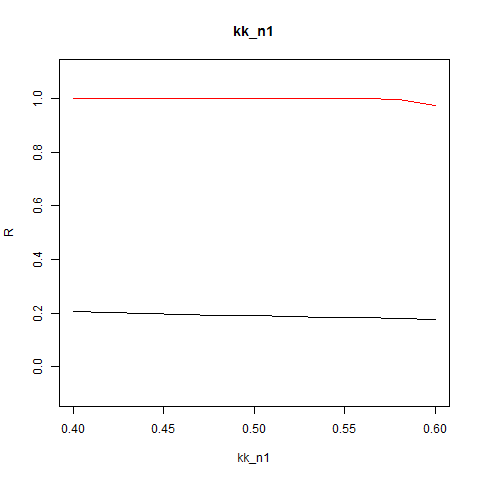 | 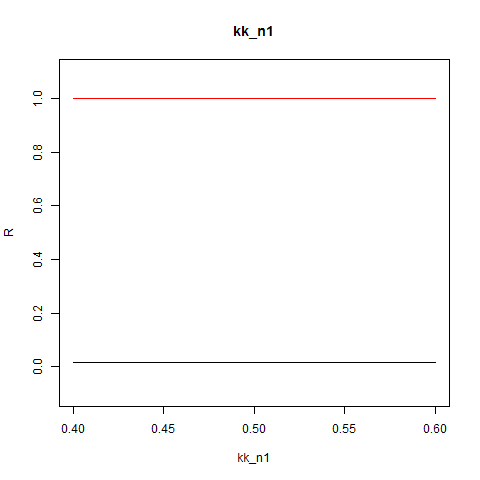 | 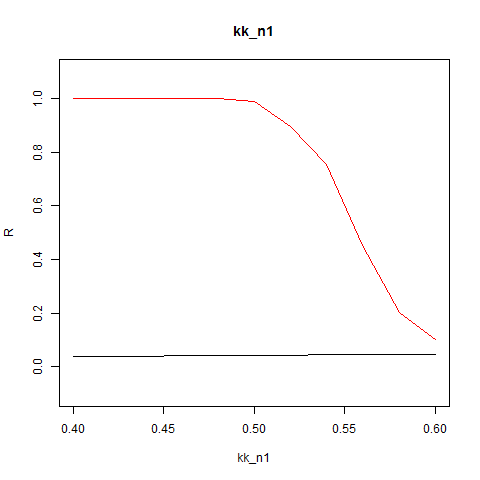 |
| dm_n1 | 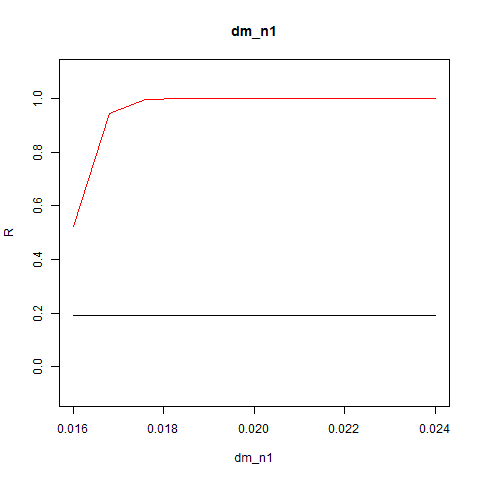 | 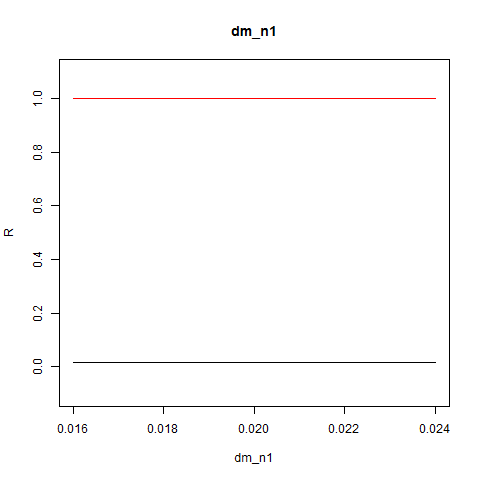 | 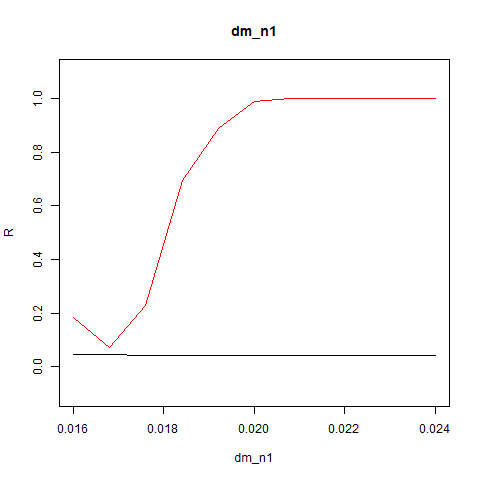 |

## Speed of Synchronization under random parameter variation

Speed of synchronization for three initial state configurations with randomly altered parameter values by +/- 20% from their default value.

Figure 2

Speed of synchronization of the two-cells system with (red solid line) and without (black solid line) cis-inhibition: the randomly drawn parameter value is displayed in the title of each plot.

|  | **Initial1** | **Initial2** | **Initial3** |
| --- | --- | --- | --- |
| K | 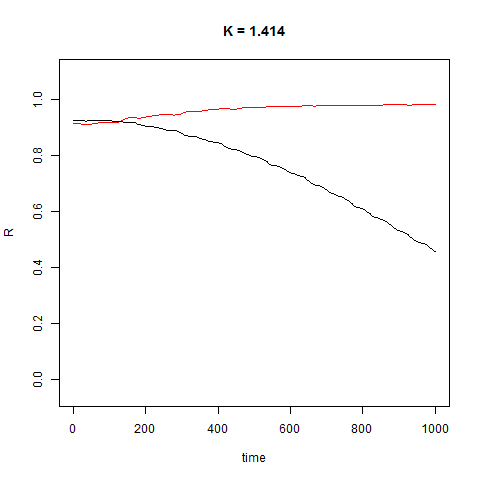 |  |  |
| dpC_h7 |  |  |  |
| G_h7 |  |  |  |
| F_h7 |  |  |  |
| epC_h7 |  |  |  |
| epN_h7 |  |  |  |
| k_h7 |  |  |  |
| dmC_h7 |  |  |  |
| dmN_h7 |  |  |  |
| emN_h7 |  |  |  |
| Hcoef_nic |  |  |  |
| Hcon_nic |  |  |  |
| r_DN |  |  |  |
| r_DNcis |  |  |  |
| dpC_nic |  |  |  |
| G_nic |  |  |  |
| F_nic |  |  |  |
| epC_nic |  |  |  |
| epN_nic |  |  |  |
| K_d1 |  |  |  |
| dpC_d1 |  |  |  |
| epC_d1 |  |  |  |
| epM_d1 |  |  |  |
| dmC_d1 |  |  |  |
| dmN_d1 |  |  |  |
| emN_d1 |  |  |  |
| kk_d1 |  |  |  |
| K_n1 |  |  |  |
| dpC_n1 |  |  |  |
| epC_n1 |  |  |  |
| dpM_n1 |  |  |  |
| kk_n1 |  |  |  |
| dm_n1 |  |  |  |

Parameter dependent oscillatory behavior

Figure 3

Influence of individual model parameters on Hes7 oscillations: displayed are the minimum and maximum amplitudes under parameter variations.

|  | **Hes7** | **Dll1** | **Notch1** | **NICD** |
| --- | --- | --- | --- | --- |
| decay rate of mRNA in cytoplasm (dmC) |  |  |  |  |
| decay rate of mRNA in nucleus (dmN) |  |  |  |  |
| decay rate of protein in cytoplasm (dpC) |  |  |  |  |
| decay rate of protein in cell membrane (dpM) |  |  |  |  |
| G |  |  |  |  |
| F |  |  |  |  |
| export rate of mRNA in nucleus (emN) |  |  |  |  |
| export rate of protein in cytoplasm (epC) |  |  |  |  |
| export rate of protein in nucleus (epN) |  |  |  |  |
| export rate of protein in cell membrane (epM) |  |  |  |  |
| translation rate (K) |  |  |  |  |
| tran - scription rate (k) |  |  |  |  |
| Hill coefficient for Hes7 promoter of Hes7 (HCoef_hes) |  |  |  |  |
| Hill coefficient for NICD promoter of Hes7 (HCoef_nic) |  |  |  |  |
| protein critical value for NICD promoter of Hes7 (HCon_nic) |  |  |  |  |

| rDN |  |
| --- | --- |
| rDNcis |  |

# References

[Garcia-Ojalvo:04]: Jordi Garcia-Ojalvo, Michael B. Elowitz, Steven H. Strogatz, Modeling a synthetic multicellular clock: Repressilators coupled by quorum sensing, PNAS 101, 2004, p 10955-10960.
