# Supplementary material for: Fast Synchronization of Ultradian Oscillators Controlled by Delta-Notch Signaling with Cis-Inhibition
Source: PLoS Comput Biol. 2014 Oct 2;10(10):e1003843. doi: 10.1371/journal.pcbi.1003843 (PMC4196275; doi:10.1371/journal.pcbi.1003843)
Supplement: Text S2 — Download information including a mini manual of the program. The file ‘HowToInstallAndRunTheProgram’ explains how to install the program on different operating systems and how to use the graphical user interface. (PDF) [file pcbi.1003843.s014.pdf]

Fast synchronization of ultradian oscillators controlled by Delta-Notch signaling with cis-inhibition

Simulation Program Mini-Manual

System Requirements

We implemented our simulation program as a Java application on a dual core processor machine using jdk1.6 and Java3D version 1.5.2. To run it we use an amount of 1500MB of the memory allocation pool (java virtual machine options: -Xms1500m -Xmx1500m).

We tested it on

- **Ubuntu 12.04.2 LTS** with
  - **Java versions "1.6.0_45" and "1.7.0_45"**
- **Microsoft Windows 7** with
  - **Java version "1.7.0"**
  - **Java 3DTM 1.5.1 Release**.
- **Mac OS X 10.8.5 and 10.9** with
  - **Java version "1.6.0_65"**
  - **Java 3DTM pre-release build 1.6.0** (see below j3d_lib/)
  - **Java™ language bindings to the OpenGL® version 2.1.5** (see below j3d_lib/).

Installation and Starting the Application

To run the application you need to install the packed file SIM13.zip, which can be downloaded from *http://www.helmholtz-muenchen.de/fileadmin/IEG/ZIP/downloads/simulation13/SIM13.zip*.

After unpacking the file you get a directory (*SIM/*) with the following configuration:

- Subdirectory *ICONS/* contains figures used to design the graphical user interface of the simulation program.
- Subdirectory *j3d_lib/* contains the java3d (resp. java bindings to OpenGL) jar files needed to make the application run under Mac OS X 10.8+ (see *SIM/README* for more details).
- Subdirectory *sim_lib/* contains all supplementary jar files used by the application.
- Subdirectory *sim_model/* contains the standard configuration files for the gene regulatory networks mentioned in the publication and the supplementary material: *Hes7_DN/configfile* corresponds to the model shown in **Figure 1** (publication), while subdirectories *Hes7_DN_Lfng/* and *Fgf8_on_Hes7_dpC/* contain configuration files for the models shown in **Figure S1** and respectively **Movies S3-S6** and **Figure 4** (supplementary material). C*onfigfile* saves the parameter values needed to start the simulation.
- Executable file *sim.jar* contains the simulation program.
- Executable batch file *startSIM.bat* starts the application on **Windows**.
- Executable shell script *startSim_linux.sh* starts the application on **Linux.**
- Executable shell script *startSim_macosx.sh* starts the application on **Mac OS X**.

Please read the file *SIM/README* for more details.

Starting the Simulation

The application provides a graphical user interface (GUI), on which the parameter values needed to start the simulation can be changed. The buttons to start and stop the simulation are on the bar at the bottom of the GUI:

- By clicking on ***'start'*** the simulation starts with the configuration status described on the parameter panel (see chapter **Using the Graphical User Interface**).
- Button ***'cancel'*** terminates the Java application.
- It is possible to return from the simulation panel to the parameter panel by clicking ***'return'***.

Using the Graphical User Interface (GUI)

The picture below shows the part of the GUI containing information about the genes building the gene regulatory network (GRN) and its interactions.

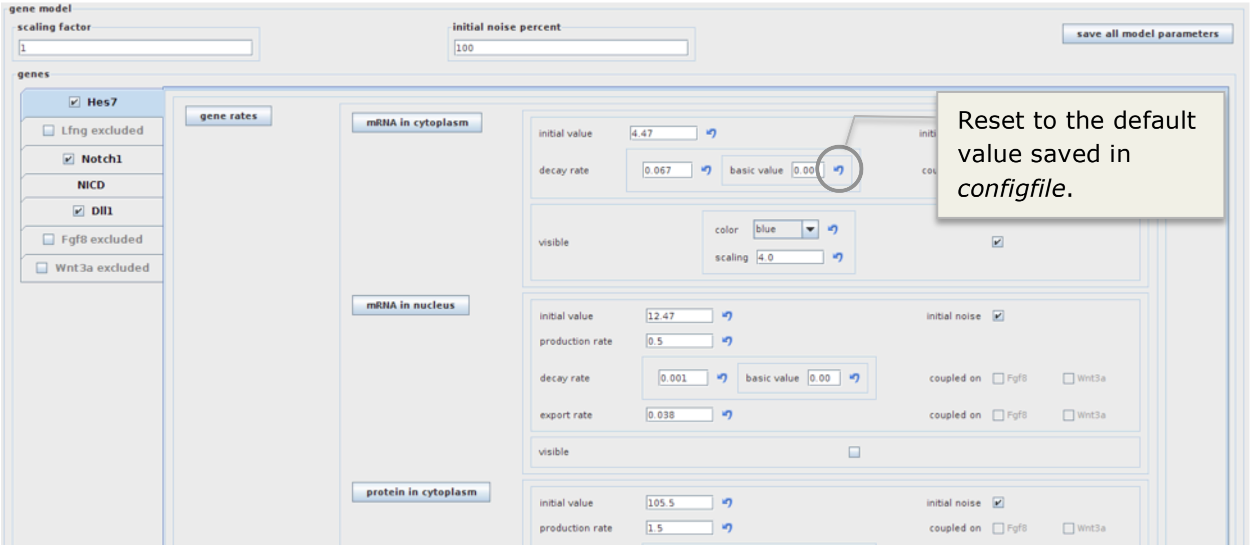

- To change the oscillation period all parameters can be scaled by the ***'scaling factor'***-value, which is equivalent to scaling by time. To do so proceed as

follows:

1. Change the value and press *ENTER* to continue.
2. A pop-up window informs you what effect the value change has. Click ***OK*** to continue or just close the window to cancel the action.

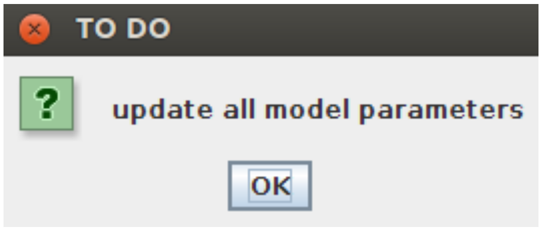

- The ***'initial noise percent'*** is a simple measure for the initial noise, i.e. by selecting the ***'initial noise'***-option we add to the initial concentration value the entered percentage of the initial value multiplied by a random value between zero and one.

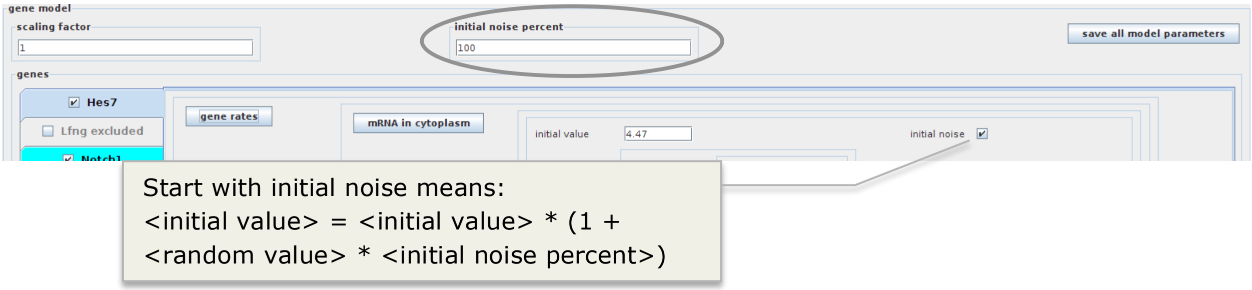

- By clicking ***'save all model parameters'*** an update of the configuration file *sim_model/<model_description>/configfile* occurs. All modifications made on

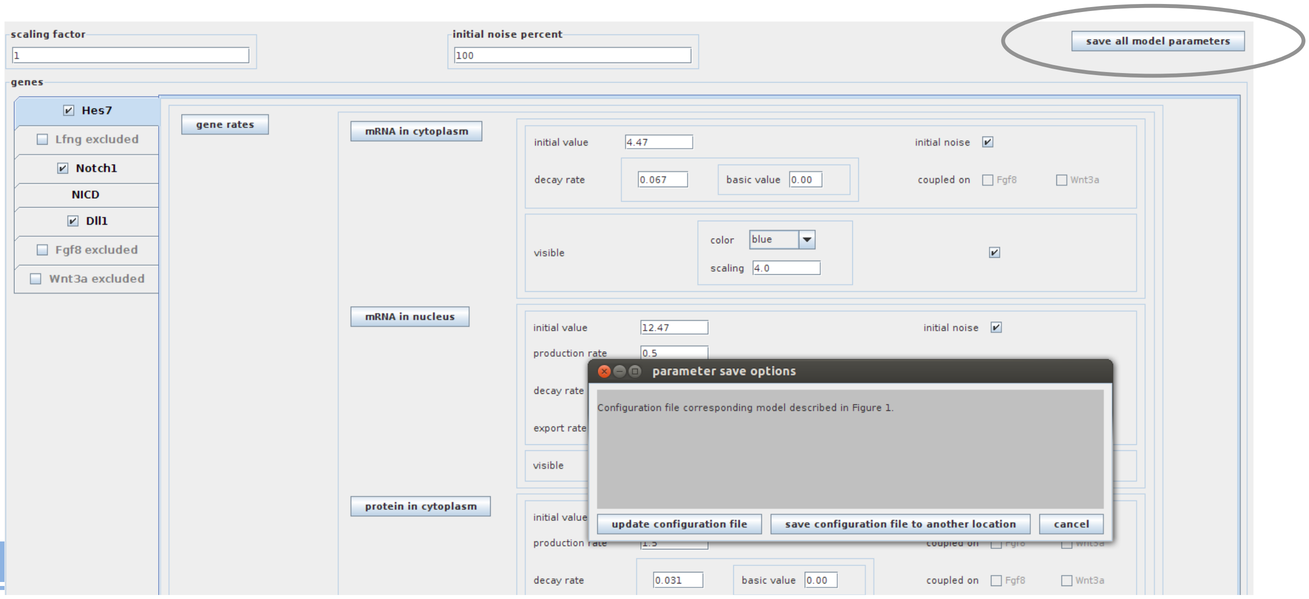

the GUI can be thus used by another program run. The old configuration file will be moved to *OLD/configfile.OLD<index>*.

- All network **genes** are described on panels sharing the same space. Selecting the corresponding tab can access the parameters of one gene. The color of the selected tab indicates for which gene the concentration values of mRNA or protein are visualized during the simulation run or whether gradient genes are coupled or not.

  Cyan color tags the gene which gene product is shown during the simulation.

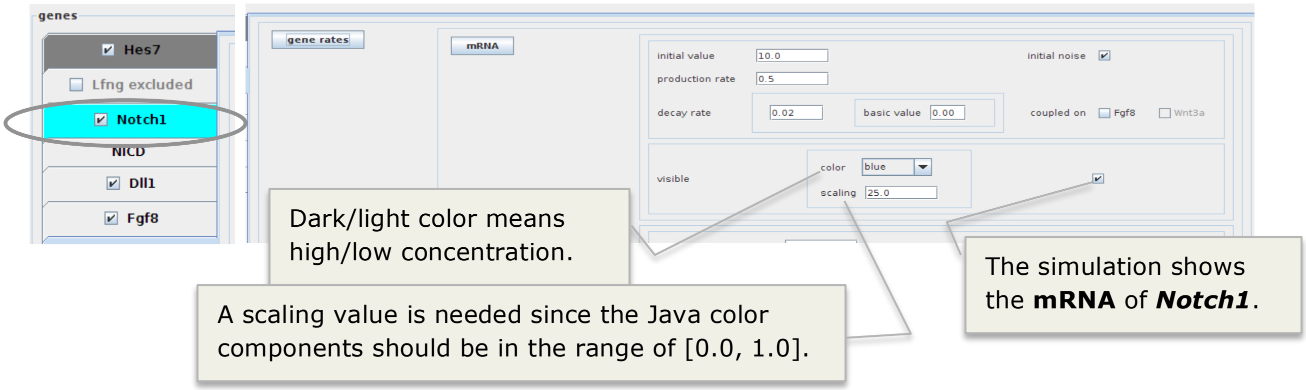

  A **dark gray** colored tab indicates that gradient genes are coupled.

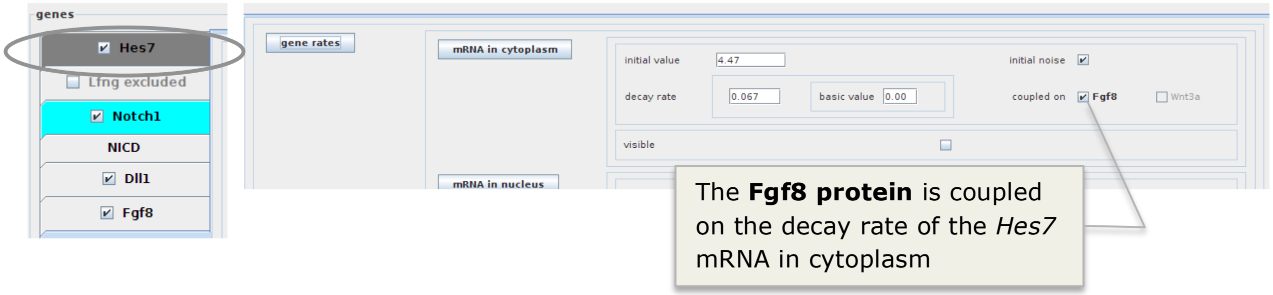

  Clicking on the corresponding check box can change the status of each gene.

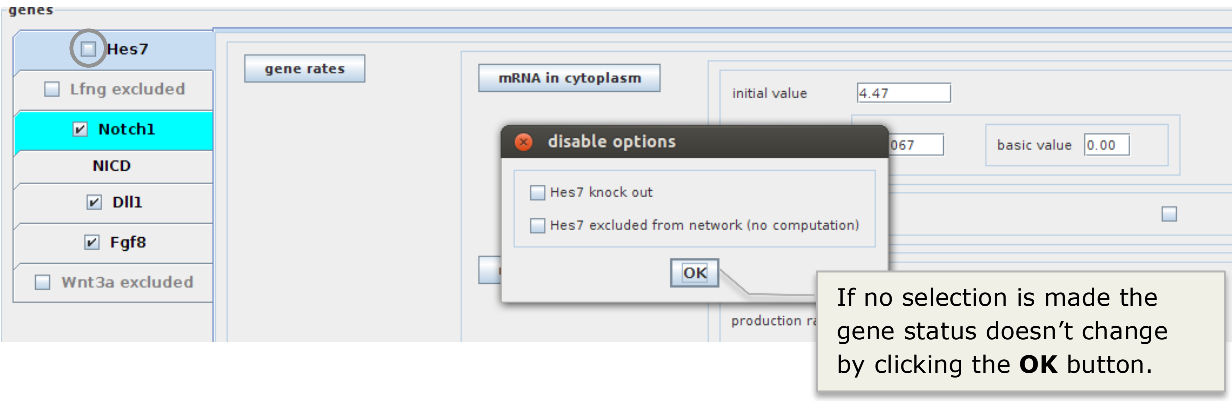

1. We simulate a **gene knock out** by setting the mRNA transcription rate to 0.
2. Gene **excluded from network** means that no computation would be done for its products. In case of Delta-Notch genes the elimination of ***Dll1*** (***Notch1***) from the GRN implies also the elimination of ***Notch1*** (***Dll1***) and **NICD**.
3. In case of ***Fgf8*** a *partial inhibition* is also supplied, which means that the protein production rate can be reduced by a defined amount at a defined time step during the simulation.

Gene panels contain also information about gene promoter.

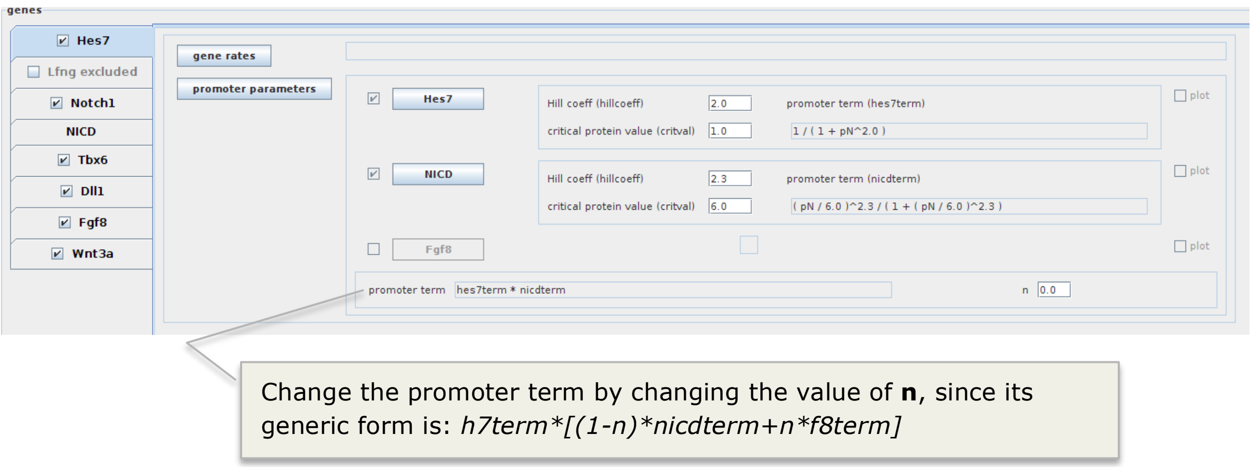

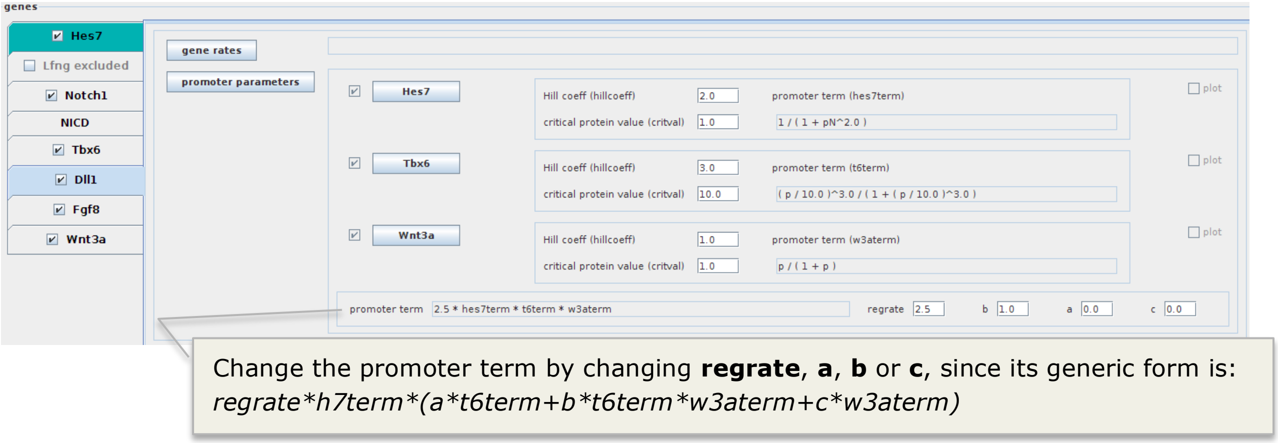

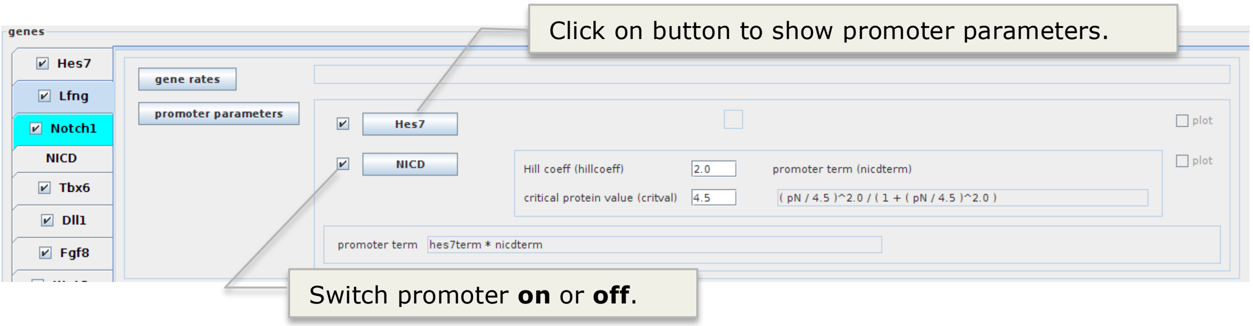

Select the ***'mitosis'*** check box to simulate **mitosis**, which is done by setting the transcription rate to 0 for the selected genes. During the simulation the cells undergoing mitosis are colored orange.

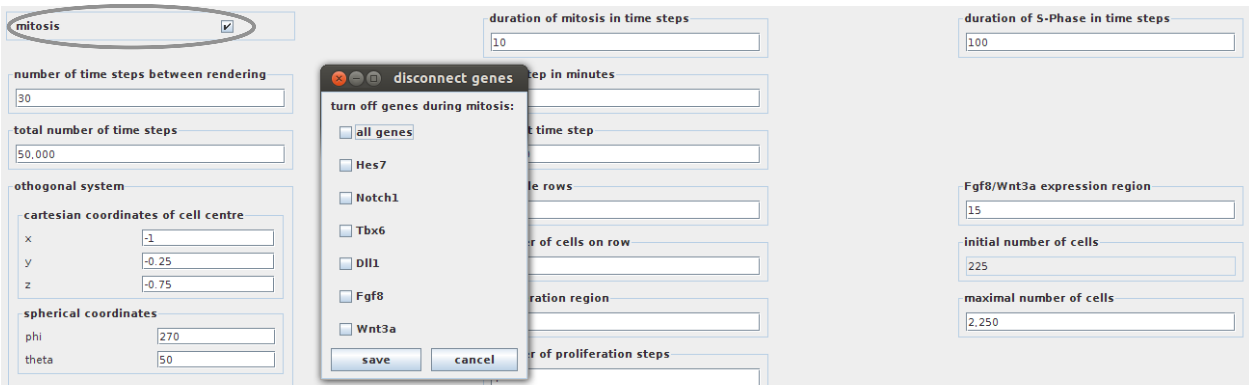

The lower part of the GUI is reserved for general settings like the layout of the proliferating cells and the way they proliferate.

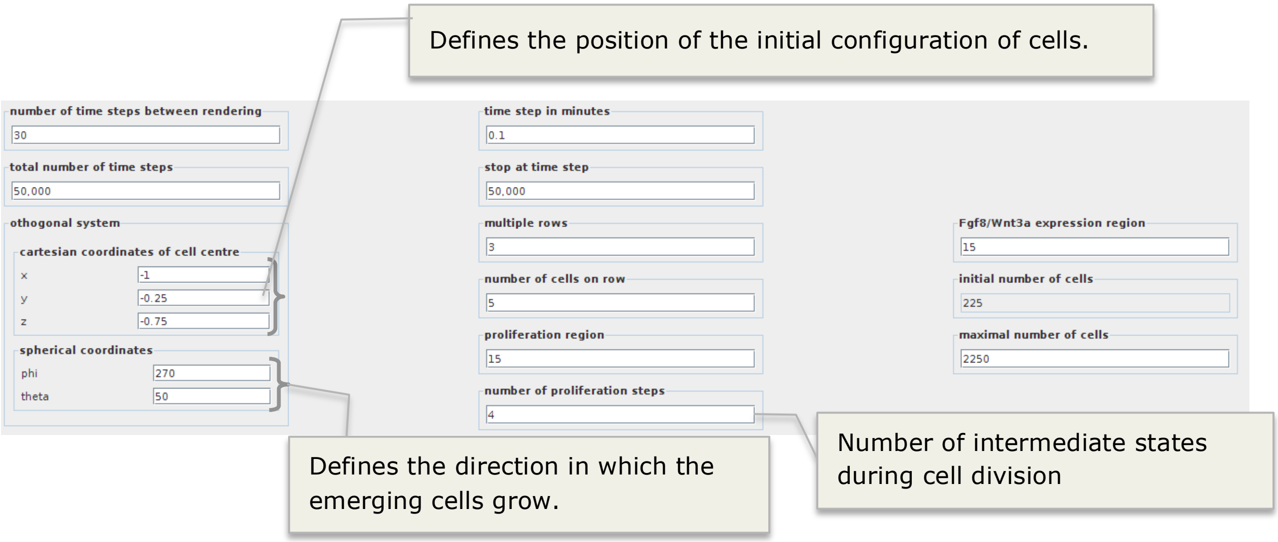
Defines the position of the initial configuration of cells.
number of time steps between rendering
30
total number of time steps
50,000
othogonal system
cartesian coordinates of cell centre
x -1
y -0.25
z -0.75
spherical coordinates
phi 270
theta 50
time step in minutes
0.1
stop at time step
50,000
multiple rows
3
number of cells on row
5
proliferation region
15
number of proliferation steps
4
Fgf8/Wnt3a expression region
15
initial number of cells
225
maximal number of cells
2250
Defines the direction in which the emerging cells grow.
Number of intermediate states during cell division

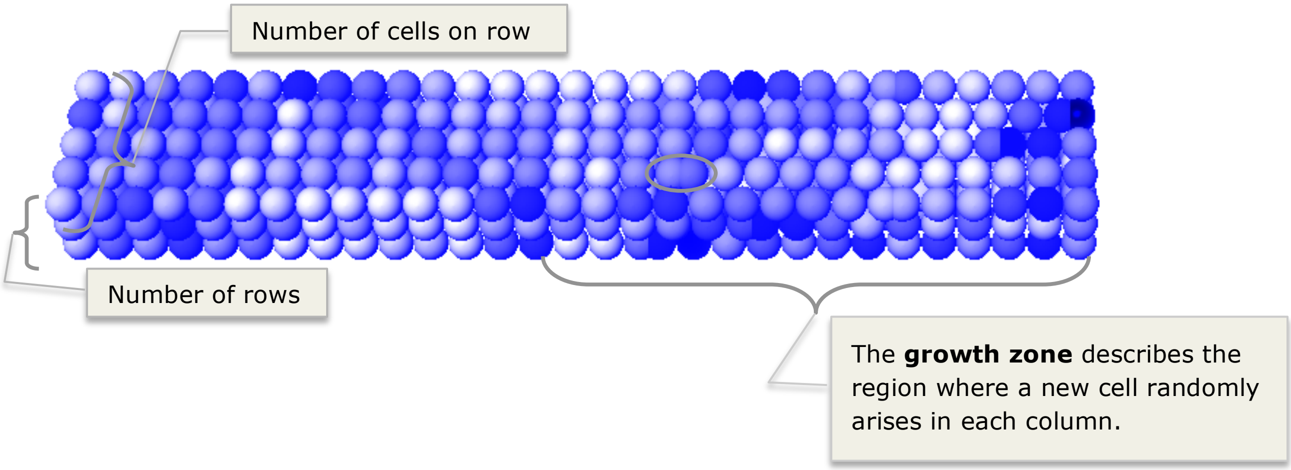
Number of cells on row
Number of rows
The **growth zone** describes the region where a new cell randomly arises in each column.

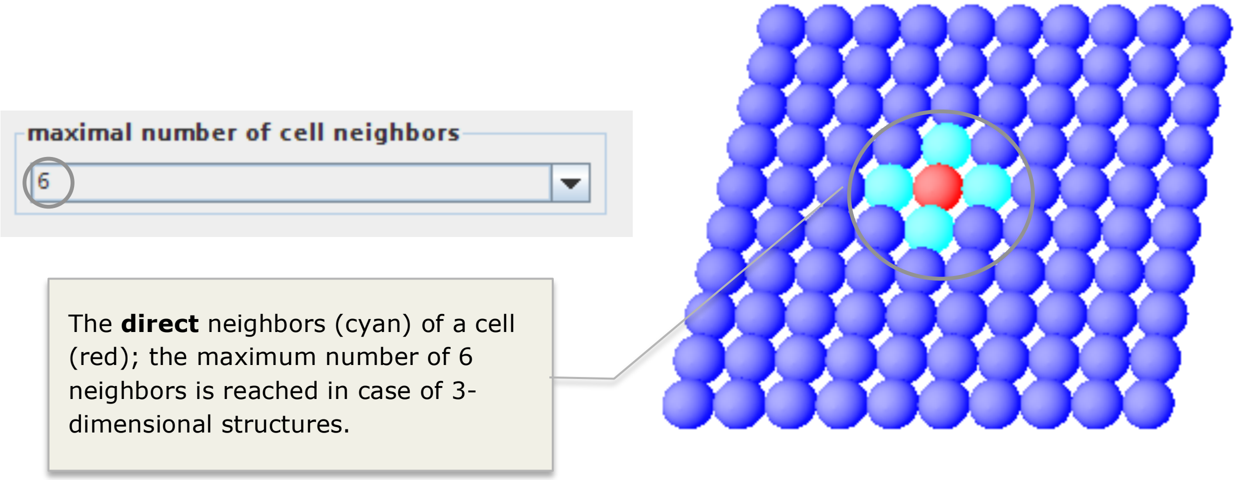
maximal number of cell neighbors
6
The **direct** neighbors (cyan) of a cell (red); the maximum number of 6 neighbors is reached in case of 3-dimensional structures.

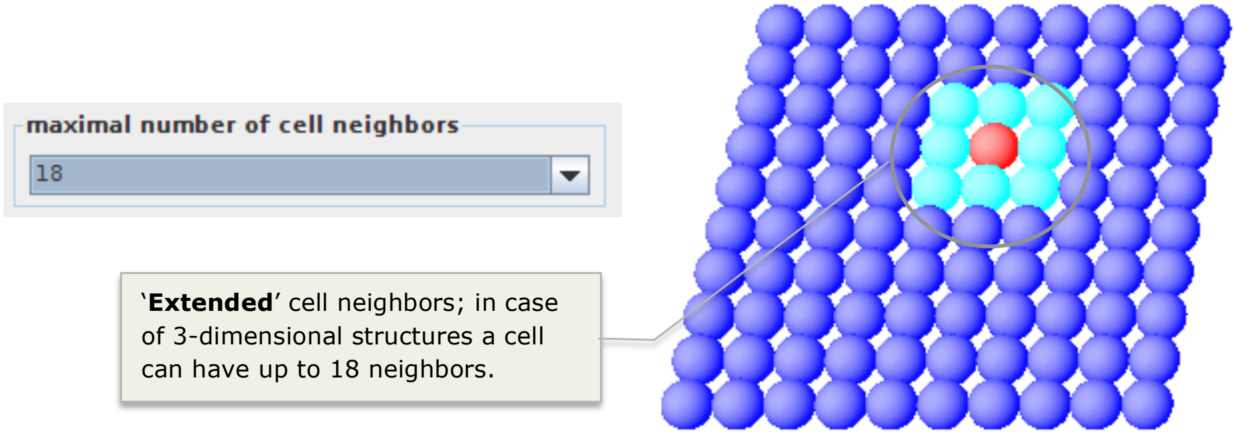

The ***'plot data for cells'*** field gives users the option to output the oscillators numerical data, which means that after each step of the *Runge-Kutta* method used to solve the system of differential equations modeling the GRN, the data will be written to a file, which can be read by an appropriate program (like *gnuplot*) to visualize the time course of the concentrations.

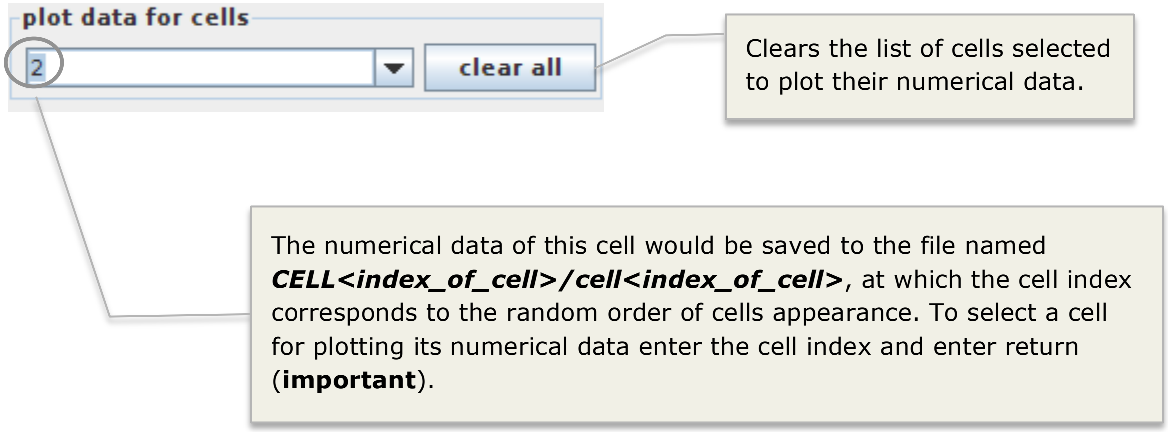

Select the ***'correlation function'*** check box to write the values of the correlation function described in the manuscript to the text file '*EVAL_DATA/correlation.dat*' and accordingly the ***'R function'*** check box for the R-synchronization-measure with the output file '*EVAL_DATA/rvalues.dat*'.

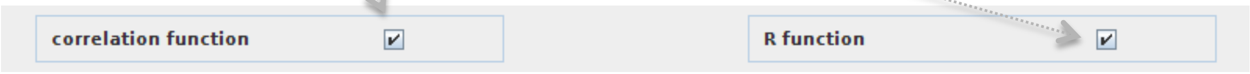

Please notice that the program execution may get very slow when both function options are selected.

As we use a simple 4th order *Runge-Kutta* Algorithm and most decay terms are linear (i.e. not saturated, we would otherwise introduce even more unknown constants) negative concentrations can be induced by choosing parameters or noise, which stray too wide from the standard values.
If negative values occur during the simulation a pop-up warning window informs the user about which cells (together with their neighbors) are involved, giving him the opportunity to return and choose another parameter values or cancel the application.
